# Selective CRAF Inhibition Elicits Transactivation

**DOI:** 10.1021/jacs.0c11958

**Published:** 2021-03-22

**Authors:** Charles W. Morgan, Ian L. Dale, Andrew P. Thomas, James Hunt, Jason W. Chin

**Affiliations:** † Medical Research Council Laboratory of Molecular Biology, Cambridge CB2 0QH, United Kingdom; ‡ Discovery Sciences, R&D, AstraZeneca, Cambridge CB4 0WG, United Kingdom; § Medicinal Chemistry, Oncology R&D, AstraZeneca, Cambridge CB4 0WG, United Kingdom; ∥ Antibody Discovery & Protein Engineering, R&D, AstraZeneca, Cambridge CB21 6GH, United Kingdom

## Abstract

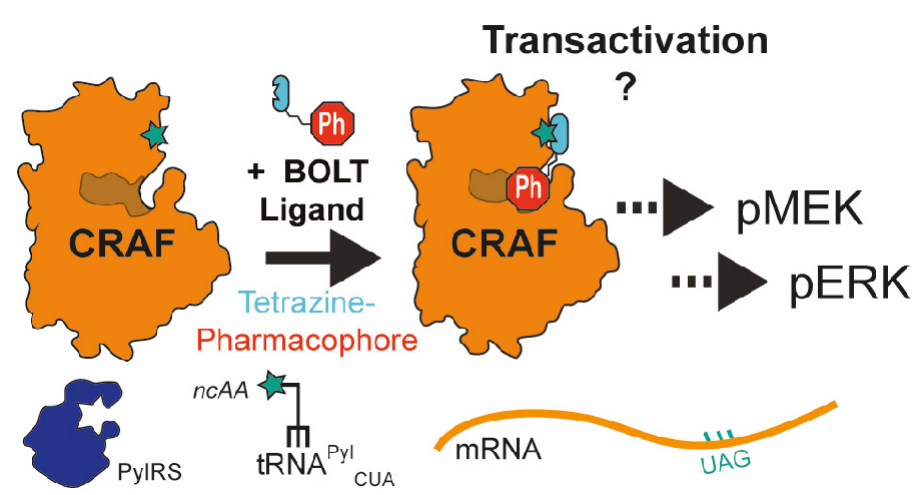

Discovering molecules that regulate closely related protein isoforms is challenging, and in many cases the consequences of isoform-specific pharmacological regulation remains unknown. RAF isoforms are commonly mutated oncogenes that serve as effector kinases in MAP kinase signaling. BRAF/CRAF heterodimers are believed to be the primary RAF signaling species, and many RAF inhibitors lead to a “paradoxical activation” of RAF kinase activity through transactivation of the CRAF protomer; this leads to resistance mechanisms and secondary tumors. It has been hypothesized that CRAF-selective inhibition might bypass paradoxical activation, but no CRAF-selective inhibitor has been reported and the consequences of pharmacologically inhibiting CRAF have remained unknown. Here, we use bio-orthogonal ligand tethering (BOLT) to selectively target inhibitors to CRAF. Our results suggest that selective CRAF inhibition promotes paradoxical activation and exemplify how BOLT may be used to triage potential targets for drug discovery before any target-selective small molecules are known.

## Introduction

Selective regulation of protein isoforms with small molecules remains an outstanding challenge. In many cases no small molecule exists that can selectively target a specific isoform and so the potential of selective pharmacological regulation remains unknown. Strategies that define the consequence of selective pharmacological regulation for specific isoforms would provide an approach for triaging molecular targets and enable efforts to be focused on developing selective small molecules for the most valuable and validated targets. However, addressing this challenge without isoform-selective small molecules in hand presents an apparent paradox. For protein kinases this paradox has been addressed by the mutation of a gatekeeper residue in the active site to create an active enzyme containing a “hole” that can be selectively inhibited by an ATP analogue containing a chemical “bump”;^1^ this principle has recently been extended to bromodomain proteins,^2^ glyco-transferases,^3^ and methyl transferases.^4^

**Figure 1:**
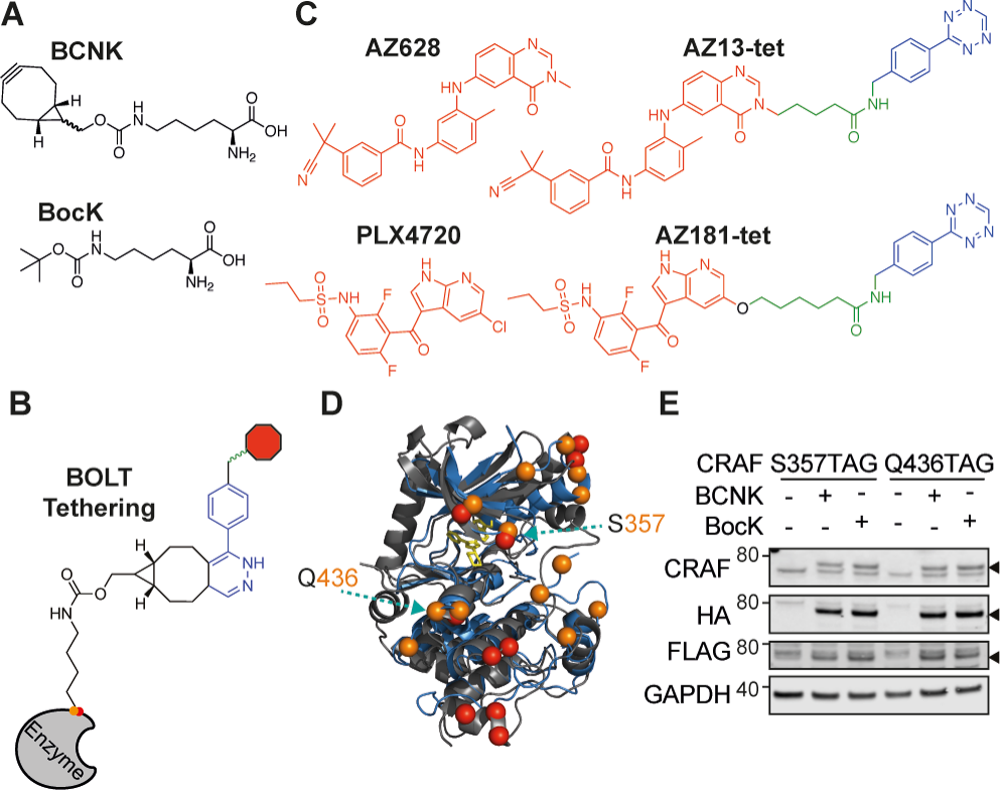
Designing BOLT ligands and sites of CRAF tethering. (A) Structures of noncanonical amino acids (ncAA) BocK and BCNK. ncAA are site-specifically incorporated into amber (UAG) variants of the CRAF gene. (B) Schematic of tethering between BOLT ligand, containing tetrazine (blue), linker (green), and pharmacophore (red), and BCNK containing proteins following an inverse electron demand Diels–Alder reaction between the BCNK and tetrazine. (C) Chemical structures of parent pharmacophores and the corresponding BOLT ligands. RAF pharmacophores, AZ628 (type II), and PLX4720 (type I) are shown in red. BOLT ligands AZ13-tet and AZ181-tet are shown; the tetrazine moiety is in blue, the linker in green, and the pharmacophore in red. (D) Structural superposition of MEK (gray) and CRAF (blue) kinase structures. A small-molecule inhibitor (yellow) occupies the ATP binding pocket. Spheres highlight positions tested for amber suppression expression and tethering, MEK (red) and CRAF (orange). Figure created using Pymol. PDB: 3ZLS MEK; 3OMV CRAF. (E) Immunoblot of the indicated CRAF variants showing full length and ncAA-dependent expression. Variants (small arrow) include C terminal epitope tags, FLAG, and HA (3xFLAG-HA) to ensure immunoprecipitation and detection of full length CRAF. Plasmids containing 4x[tRNAPyl] and CRAF(S357TAG) or CRAF(Q436TAG) variants were transfected into HCT116* cells. Cells were grown with indicated ncAA (2 mM BocK, 200 μM BCNK). Lysates were collected after 48 h of expression. Extended screening and testing of CRAF(YXXXTAG) alleles are available in Figure S3; XXX indicates the position at which the codon for a canonical amino acid (Y) is replaced with the amber codon (TAG).

We previously described a distinct approach for the selective regulation of protein isoforms named *b*i*o*rthogonal *l*igand *t*ethering (BOLT). In this approach we site-specifically and cotranslationally encode a noncanonical amino acid (ncAA) bearing a bio-orthogonal group (commonly (2*S*)-2-amino-6-((((1*R*,8*S*)-bicyclo[6.1.0]non-4-yn-9-ylmethoxy)carbonyl) amino) hexanoic acid (BCNK (Figure 1A)) or Nε-(((2-methylcycloprop-2-en-1-yl)methoxy)carbonyl)- l-lysine (CypK)^5,6^ into the target protein using genetic code expansion.^7,8^ We then add a druglike small molecule–tetrazine conjugate to cells. The conjugate reacts with the target protein through a rapid bioorthgonal inverse electron demand Diels–Alder reaction^9−12^ and tethers the druglike small molecule to the target (Figure 1B). Such tethering can increase the effective concentration of the ligand in proximity of the active site^13−19^ and lead to selective regulation of the target protein. We have previously shown that this approach enables the selective inhibition of MEK1 or MEK2, and by using a photoswitchable linker in the druglike small molecule–tetrazine conjugate, we have demonstrated reversible photocontrol of MEK1; we have also extended the approach to LCK.^20^ Because the ligand is tethered at sites distinct from the active site, BOLT does not require mutation of conserved residues in the active site, which can abrogate the functions of many enzymes. Moreover, sites of tethering distal from the active site, which do not affect protein function, can be simply found and transferred between similar proteins.

The Ras/ERK signaling pathway controls many cellular processes, including differentiation, proliferation, and survival. Frequent mutations in the pathway, primarily generating activated forms of RAS and BRAF, are observed in more than 30% of human cancers.^21,22^ The canonical pathway integrates extracellular signals through transmembrane receptors, switches Ras GTPase to the active GTP-bound form, and recruits RAF kinases to the membrane, where they are activated.^23^ Three RAF protein kinase (A, B, and C) serve as effector kinases in the RAS-ERK signaling cascade. These kinases drive the activating phosphorylation of MEK, which ultimately results in an ERK-mediated transcriptional response.^24^ Key to the activation of RAF kinases is the RAS-mediated disruption of their autoinhibited conformation^25^ and the formation of homo- and heterodimers.^26−28^

A great deal of effort has gone into generating RAF inhibitors. In cells expressing mutant BRAF (e.g., V600E), inhibitors suppress RAF activity and ERK signaling, while in cells expressing wild-type BRAF most inhibitors cause an undesired increase in RAF activity and ERK signaling (so-called “paradoxical activation”).^29−32^ Understanding the specific mechanism of action of RAF inhibitors has been the focus of intense research efforts and has challenged the academic and drug discovery communities for nearly 2 decades.^33−38^

Prevailing models of paradoxical activation center on inhibitors promoting RAF dimerization and ultimately eliciting MEK-ERK pathway activation, an outcome that is amplified by oncogenic RAS mutations.^37^ The role of RAF dimerization is central to both physiological and inhibitor induced signaling.^23,39^ The homo- and heterodimers formed by wild-type BRAF and CRAF are responsible for phosphorylating MEK. While mutant BRAF^V600E^ is constitutively active and has a limited role in dimerization,^40^ the BRAF-CRAF heterodimer is believed to be the primary species in both native signaling and paradoxical activation.^39,41,42^ Genetic and biochemical results have repeatedly implicated CRAF as the primary species responsible for phosphorylating MEK in paradoxical activation and native signaling.^30−32,43−46^ Specifically, inhibitor-bound BRAF is implicated in promoting heterodimerization with unbound CRAF, causing transactivation of CRAF through an allosteric mechanism at the protein–protein interface between protomer kinase domains.^30,32^

Decades of genetics research have employed both germline and conditional allele manipulations of RAF isoforms; these studies have revealed both redundancy and distinct functions for BRAF and CRAF in different cell types and stages of cancer progression.^47−50^ Recent genetic ablation of CRAF suggested that removal of the protein may afford a therapeutic benefit.^45,51^ However, as no well-characterized CRAF-selective inhibitors have been reported, the consequences of selective CRAF inhibition have remained unknown. Here, we develop BOLT to selectively target inhibitors to CRAF. Our results suggest that selective CRAF inhibition promotes paradoxical activation.

## Results and Discussion

There are over 2 dozen well-characterized small-molecule inhibitors targeting RAF kinases, with characterization spanning *in vitro*, preclinical, and clinical studies.^37^ Notably, none are selective to the CRAF isoform in mutant RAS cells. Drawing upon available RAF-selective pharmacophores, we designed and synthesized a series of potential BOLT ligands composed of three chemical moieties: a pharmacophore, a linker, and a tetrazine (Figure 1C and Supporting Information). Pharmacophores were chosen from classic and distinct RAF inhibitors, PLX4720 (type I, αC-OUT/DFG-IN) and AZ628 (type II, αC-IN/DFG-OUT). We created AZ13-tet (containing the AZ628 pharmacophore) and the AZ181-tet (containing the PLX4720 pharmacophore). We used the structure of RAF-inhibitor complexes^52,53^ to design synthetically accessible BOLT ligands that should not interfere with pharmacophore binding. We demonstrated that BOLT ligands exhibited similar cellular responses and paradoxical activation to their parent inhibitors; as expected, BOLT ligands showed a decrease in potency (Figure S1).

We chose sites for ncAA incorporation in CRAF based on the structure of RAF, a structural alignment of its kinase domain with that of MEK, and previous work developing BOLT on MEK (Figure 1D and Figure S2).^20^ We encoded BCNK at sites in both the N and C lobes of CRAF using the *Mm*PylRS(Y306A, Y384F)^54^ /*Mm*tRNA^Pyl^_CUA_ pair, BCNK, and CRAF (YXXXTAG) (Figure S3A,B). The majority of selected positions showed BCNK-dependent expression of full length CRAF and were efficiently labeled with a fluorescent tetrazine conjugate (Figure S3F). We also incorporated BocK (Figure 1A), an amino acid containing a nonreactive side chain.

We chose CRAF(S357TAG) and CRAF(Q436TAG) (in the N lobe and C lobe of the kinase, respectively) for further characterization; these mutants produce good levels of ncAA-dependent expression and position the ncAA, adjacent to the solvent channel, where tethering may enable the pharmacophores to access their native binding sites within the same monomer. Distance measurements suggest that tethering across the RAF dimer – in a trans mode– is prevented by the relatively short linker. Analogous positions on ARAF and BRAF were also amenable to ncAA incorporation (Figure S4).

The greatest potential utility of selective CRAF inhibition would be in tumors containing activating RAS mutations; additionally, the phenomenon of paradoxical RAF activation by current RAF inhibitors is amplified in cells containing activated RAS. We therefore engineered HCT116 (KRASG13D) cells with genetic code expansion machinery to enable the site-specific incorporation of either BCNK or BocK. A dual expression cassette encoding *Mm*tRNA^Pyl^_CUA_ and *Mm*PylRS(Y306A, Y384F) as a BFP-T2A fusion was assembled and integrated into the genome of HCT116 cells using the PiggyBac Transposase (Figure S5A).^55^ We used FACS selection for BFP fluorescence to discover clones expressing a high level of the synthetase. We thus created the stable cell line: HCT116 4x[*Mm*tRNA^Pyl^ ]*-*BFP-2A-PylRS(Y306A, Y384F) (henceforth designated HCT116*) (Figure S4B–D). We transfected 4x[*Mm*tRNA^Pyl^] CRAF(YXXXTAG) into this cell line and supplemented cells with either BCNK or BocK (Figure 1E). CRAF expression was ncAA-dependent and efficient, and we used this system to investigate the cellular consequence of BOLT ligands on RAF kinases in all subsequent experiments. In all experiments, we distinguished the effects of ligand tethering (BOLT) from the effects of reversible ligand binding to all RAF species using matched controls in which BocK (which does not react with tetrazines) replaced BCNK in CRAF.

**Figure 2:**
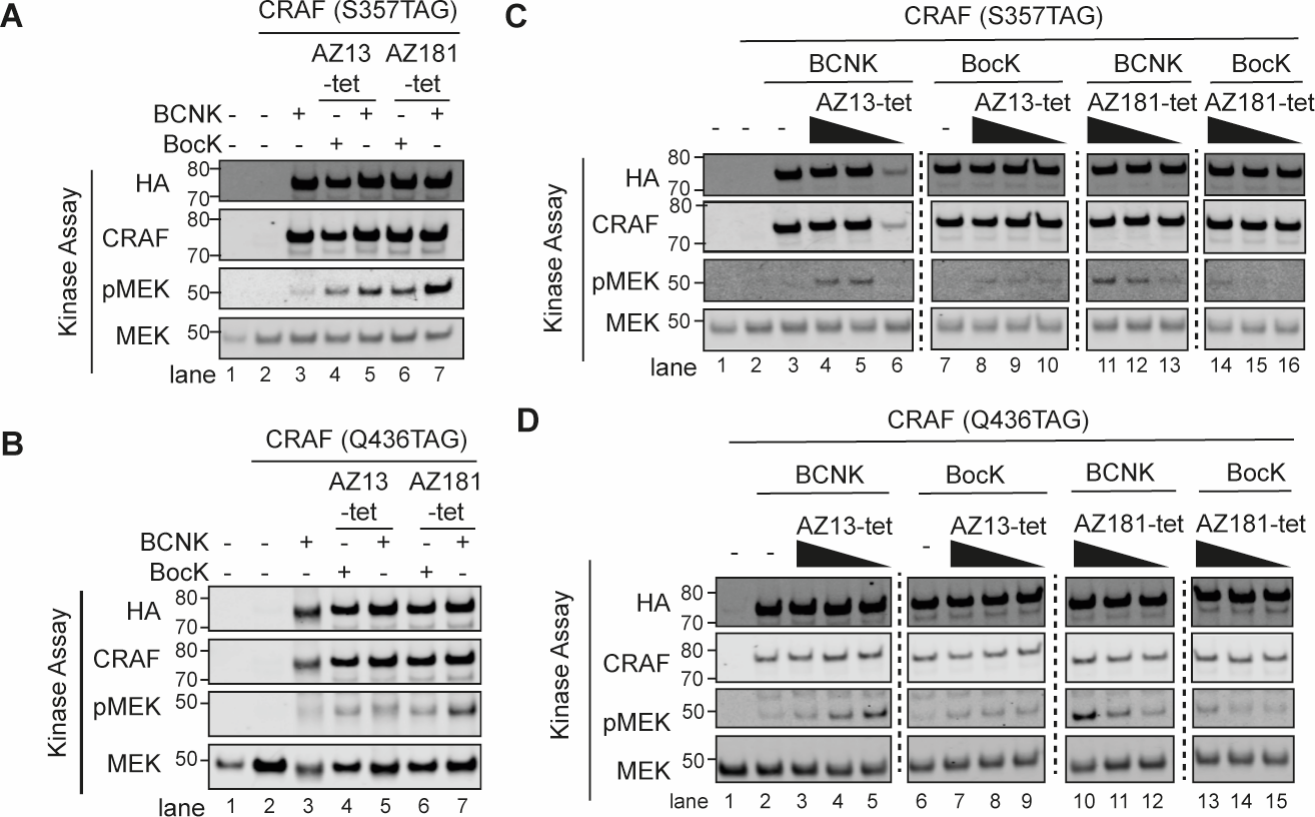
BOLT of CRAF variants elicits kinase activation. (A) Immunoblot of kinase assays using CRAF (S357TAG) expressed with BCNK or BocK in HCT116*. Cells were washed to remove excess ncAA and then treated with indicated BOLT ligand (AZ13-tet or AZ181-tet, 2 μM). CRAF(S357BCNK) or CRAF(S357BocK) were immunoprecipitated via their C-terminal FLAG tag. The immunoprecipitate was assayed for RAF kinase activity in vitro, using catalytically dead MEK1 as a substrate. All CRAF amber alleles are HA tagged. (B) Immunoblot of kinase assays using CRAF (Q436TAG) expressed with BCNK or BocK in HCT116*. Cells were washed to remove excess ncAA and then treated with indicated BOLT ligand (AZ13-tet or AZ181-tet, 2 μM). CRAF(Q436BCNK) or CRAF(Q436BocK) were immunoprecipitated via their C-terminal FLAG tag. The immunoprecipitate was assayed for RAF kinase activity in vitro, using catalytically dead MEK1 as a substrate. All CRAF amber alleles are HA tagged. (C) Immunoblot of kinase assays using CRAF (S357TAG) expressed with BCNK or BocK in HCT116* treated with varying concentrations of BOLT ligands. The experiment was performed as described in panel A. Ligand concentrations were 2000, 200, and 20 nM. (D) Immunoblot of kinase assays using CRAF (Q436TAG) expressed with BCNK or BocK in HCT116* treated with varying concentrations of BOLT ligands. The experiment was performed as described in panel B. Ligand concentrations were 2000, 200, and 20 nM.

To investigate the consequence of selective CRAF inhibition, we expressed CRAF containing BCNK or BocK at position S357 or Q436 in HCT116* cells. We washed cells to remove the free ncAA and then added 2 μM AZ13-tet or AZ181-tet to cells for 2 h. ncAA-containing CRAF was then immunoprecipitated via a C-terminal 3xFLAG epitope tag and the eluted material was immediately assayed for RAF kinase activity, using kinase-dead, recombinant MEK1 as a substrate (Figure 2A,B and Figure S6A,B). CRAF (S357BCNK) showed an increase in MEK phosphorylation with respect to CRAF (S357BocK) in this assay when both proteins were immunoprecipitated from cells to which AZ181-tet had been added (Figure 2A). Similarly, CRAF (Q436BCNK) showed an increase in MEK phosphorylation with respect to CRAF (Q436BocK), when both proteins were immunoprecipitated from cells to which AZ181-tet had been added (Figure 2B). CRAF (S357BCNK) showed an increase in MEK phosphorylation with respect to CRAF (S357BocK) when both proteins were immunoprecipitated from cells to which AZ13-tet had been added (Figure 2A). However, CRAF (Q436BCNK) and CRAF (Q436BocK) led to comparably low levels of MEK phosphorylation, when both proteins were immunoprecipitated from cells to which AZ13-tet had been added.

Next, we repeated the experiments described above using AZ13-tet or AZ181-tet at concentrations spanning 3 orders of magnitude (20 nM, 200 nM, and 2 μM). Crucially, we observe a dose-dependent increase in MEK phosphorylation for CRAF (S357BCNK), but not CRAF (S357BocK), with both AZ13-tet and AZ181-tet (Figure 2C, Figure S6C). Notably, we observe a similar dose-dependent increase in MEK phosphorylation for CRAF (Q436BCNK) but not CRAF (Q436BocK), with AZ181-tet (Figure 2D, Figure S6D). We observe an increase in MEK phosphorylation within 20 min of ligand addition (Figure S7). Interestingly, CRAF (Q436BCNK), but not CRAF (Q436BocK), shows a high level of MEK phosphorylation, after immunoprecipitation from cells treated with 20 nM AZ13-tet, but higher concentrations of AZ13-tet decrease the MEK phosphorylation mediated by CRAF (Q436BCNK) without affecting the MEK phosphorylation mediated by CRAF (Q436BocK); the level of MEK phosphorylation mediated by CRAF (Q436BCNK) from cells treated with 2 μM AZ13-tet is low and comparable to that mediated by CRAF (Q436BocK) treated with AZ13-tet (from 20 nM to 2 μM) (Figure 2B,D).

Our data are consistent with selective CRAF inhibition, via bio-orthogonal ligand tethering, leading to transactivation of associated RAF monomers in a RAF dimer (paradoxical activation). For all of the ncAA position/BOLT ligand combinations tested, the activation is dependent on BOLT ligand concentration when the protein contains BCNK, but does not show the same level of activation when the protein does not contain BCNK. This indicates that the observed paradoxical activation is selective for CRAF and is dependent on the tethering of the ligand to CRAF. For CRAF (Q436BCNK) with AZ13-tet this activation occurs at lower concentrations of BOLT ligand and at higher concentrations we see inhibition; this is consistent with the second RAF protomer in a dimer being occupied by a ligand, and with the expected bell-shaped activity concentration curve for such systems.^30,32^ We hypothesized that when CRAF (S357BCNK) and CRAF (Q436BCNK) are immunoprecipitated, they may coimmunoprecipitate wild-type RAF species. We demonstrated that kinase-dead CRAF variants, (D486V, S357BCNK), (D486V, Q436BCNK), (A490T, S357BCNK), (A490T, Q436BCNK),^56,57^ transactivate associated RAF monomers in the presence of BOLT ligands; the matched BocK controls did not lead to transactivation (Figure S8). This strongly suggested that associated RAF dimers are responsible for the tethering-dependent paradoxical activation we observe.

Next, we aimed to confirm that wild-type RAF species can be copurified in our immunoprecipitations. AZ628, like many RAF inhibitors, is known to promote and stabilize RAF dimers in immunoprecipitations and have pronounced effects in wild-type RAF or mutant Ras cells.^30,31^ Upon immunoprecipitating CRAF (S357BCNK) or CRAF (Q436BCNK) from cells coexpressing BRAF and CRAF nanoluciferase fusions (BRAFnLuc and CRAFnLuc), we coimmunoprecipitated both exogenous RAF species; specifically, we detected increasing amounts of BRAFnluc with increasing concentrations of AZ13-tet (Figure S9). Control experiments using the matched BocK controls suggest this coimmunoprecipitation is stimulated by ligand tethering. These experiments suggest that wild-type RAF species can be coimmunoprecipitated with immunoprecipitated CRAF. Thus, the paradoxical activation we observe for CRAF (S357BCNK) or CRAF (Q436BCNK) in our BOLT experiments may result from the dimerization and transactivation of uninhibited wild-type RAF species.

**Figure 3:**
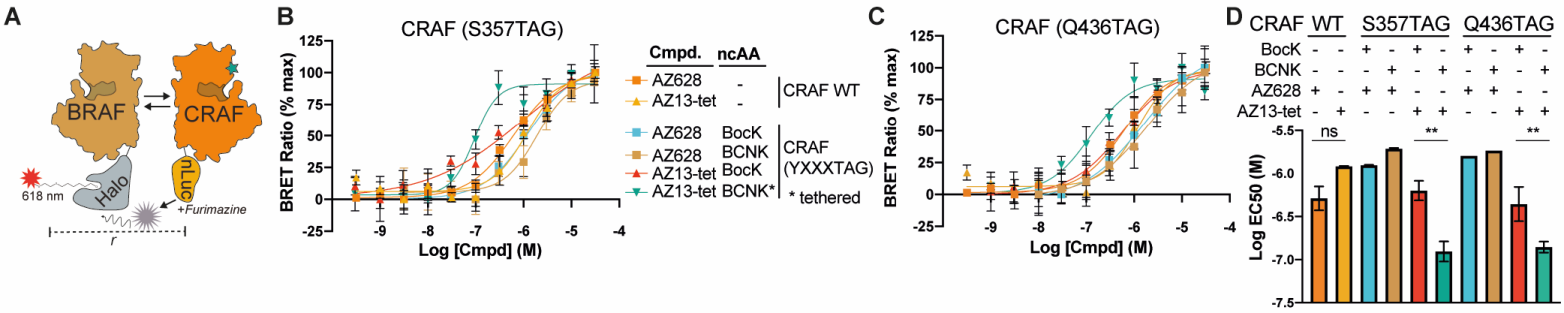
Dimerization of cellular RAF using BOLT. (A) Cellular RAF dimerization assay based on the BRET donor nanoluciferase(nLuc) and BRET acceptor chloroalkane conjugate and Halo-tagged (HT) species of BRAF kinases. Addition of nLuc substrate, furimazine, results in a dimerization-dependent energy transfer, as detected by emission of the fluorescent-dye chloroalkane conjugate. (B) Heterodimizeration of CRAFnLuc variants (wild-type and CRAF(S357TAG) incorporating BCNK or BocK) in response to an increasing concentration of AZ628 and AZ13-tet. Error bars correspond to standard deviation across four biological replicates. The continuous line corresponds to nonlinear regression (four variable) completed using Prism software. Data are color-coded as shown in the associated table. (C) Heterodimizeration of CRAFnLuc variants (wild-type and CRAF(Q436TAG) incorporating BCNK or BocK) in response to increasing concentrations of AZ628 and AZ13-tet. Error bars correspond to standard deviation across four biological replicates. Continuous line corresponds to nonlinear regression (four variable) completed using Prism software. Data are color-coded as in panel B. (D) Summary of calculated apparent EC_50_ values across the different CRAF variants and ligands, mean values shown with error bars representing standard deviation between at least two independent dose response experiments. Error bars correspond to standard deviation between calculated EC_50_ values based on nonlinear regression (four variable) modeling. Calculated EC_50_ values with confidence intervals shown in Figure S11.

We used a nanoBRET assay to further investigate BOLT-induced dimerization of CRAF in cells.^58^ RAF species were expressed with a Halo tag and CRAF (WT or variants containing BCNK or BocK) were expressed with a C-terminal nanoluciferase fusion (Figure 3A). Since AZ628 is a potent inducer of RAF dimers, we focused our experiments on characterizing AZ628 and its BOLT derivative, AZ13-tet. We observed a clear increase in dimerization of CRAF (S357BCNK) and CRAF (Q436BCNK) when cells were treated with AZ13-tet. This increase was not observed for CRAF containing BocK in place of BCNK, WT CRAF, or upon treatment with AZ628 (Figure 3B–D). We conclude that tethering of AZ-13-tet to CRAF (S357BCNK) and CRAF (Q436BCNK) leads to an increase in heterodimerization with BRAF in cells. Parallel experiments examining CRAF homodimerization, utilizing a CRAF-HaloTag variant, revealed a similar tethering-dependent increase in dimerization (Figure S10).

For S357BCNK we observe a notably steep BRET signal versus AZ13-tet concentration curve. This corresponds to a Hill slope coefficient (*n*_H_) of greater than 1. While many factors impact the Hill slope coefficient, values greater than 1 can indicate covalent and/or bivalent cooperative binding.^59,60^ Average calculated EC_50_ values from independent dose response experiments show a clear increase in potency under BOLT conditions (Figure 3D). Regression curve parameters and confidence intervals across independent experiments suggest EC_50_ values for untethered conditions are best interpreted as an upper limit of ligand potency, that is, >EC_50_ (Figure S11). In summary, BOLT led to at least a fourfold decrease in the EC_50_ for RAF dimerization. Our activity and dimerization data are consistent with selective inhibition of CRAF, leading to enhanced dimerization of other RAF monomers, and the activation of those monomers to phosphorylate MEK1.

## Conclusions

The development of small-molecule inhibitors targeting RAF protein kinases has driven advances in biomedical research and delivered drugs for the treatment of mutant BRAF-driven melanomas, which have improved patient outcomes. However, initial clinical success has been tempered by the emergence of resistance mechanisms and development of secondary tumors resulting from “paradoxical activation”.^61^ A decade of research has since deepened our understanding of the unusual pharmacology and complex regulatory mechanisms governing RAF biology and MAPK signaling.^37,53,62−66^ While our improved molecular understanding of RAF transactivation has guided patient treatment selection and influenced treatments targeting two or more nodes in the MAPK pathway,^61,62^ progress in developing RAF inhibitors effective against mutant RAS-driven tumors remains an unsolved challenge. Recent research has indicated the potential for selective inhibition of CRAF-driven signaling to be a more effective and tolerated therapeutic approach for mutant RAS tumors.^45,51^ Identifying selective CRAF kinase inhibitors that would block BRAF-CRAF heterodimer signaling could thus be a valuable drug for the treatment of mutant RAS-driven tumors, but would present a major challenge for medicinal chemistry. Before embarking on such a challenging drug discovery goal, it would be desirable to have greater confidence and confirmation that such an approach was valid and that selective CRAF inhibitors would not suffer the same problem of paradoxical activation shown by previous RAF inhibitors.

We have used BOLT to selectively tether inhibitors to CRAF and interrogate the consequences of selective CRAF inhibition. Our results suggest that selective CRAF inhibition will not be spared the liabilities observed with BRAF-selective and pan RAF inhibitors in eliciting transactivation. We suggest that future CRAF-selective pharmacophores may benefit from considering strategies taken for paradox breaking mutant BRAF inhibitors.^67^ Our results exemplify how BOLT may be used to triage potential targets for drug discovery before any target-selective small molecules are known.

## Supporting Information Available

Figures S1–S11; experimental details (PDF)
