# Supplementary material for: Selective CRAF Inhibition Elicits Transactivation
Source: J Am Chem Soc. 2021 Mar 22;143(12):4600–6. doi: 10.1021/jacs.0c11958 (PMC8041278; doi:10.1021/jacs.0c11958)
Supplement: Supplementary file 1 — ja0c11958_si_001.pdf [file ja0c11958_si_001.pdf]

SUPPORTING INFORMATION FOR:

SELECTIVE CRAF INHIBITION ELICITS TRANSACTIVATION

Charles W. Morgan[1*], Ian L. Dale[2], Andrew P. Thomas[3], James Hunt[4], Jason W. Chin[1*]

[1]Medical Research Council Laboratory of Molecular Biology, Cambridge, CB2 0QH

[2]Discovery Sciences, R&D, AstraZeneca, Cambridge, U.K.

[3]Medicinal Chemistry, Oncology R&D, AstraZeneca, Cambridge, U.K.

[4]Antibody Discovery and Protein Engineering, R&D, AstraZeneca, Cambridge, U.K

DOI: 10.1021/jacs.0c11958

TABLE OF CONTENTS

SUPPLEMENTARY FIGURES ……………………………………………………..….S2

MATERIALS AND METHODS……………………………………………………..…….S14

SYNTHETIC PROCEDURES……………………………………………………..……S18

Supplementary Figures:

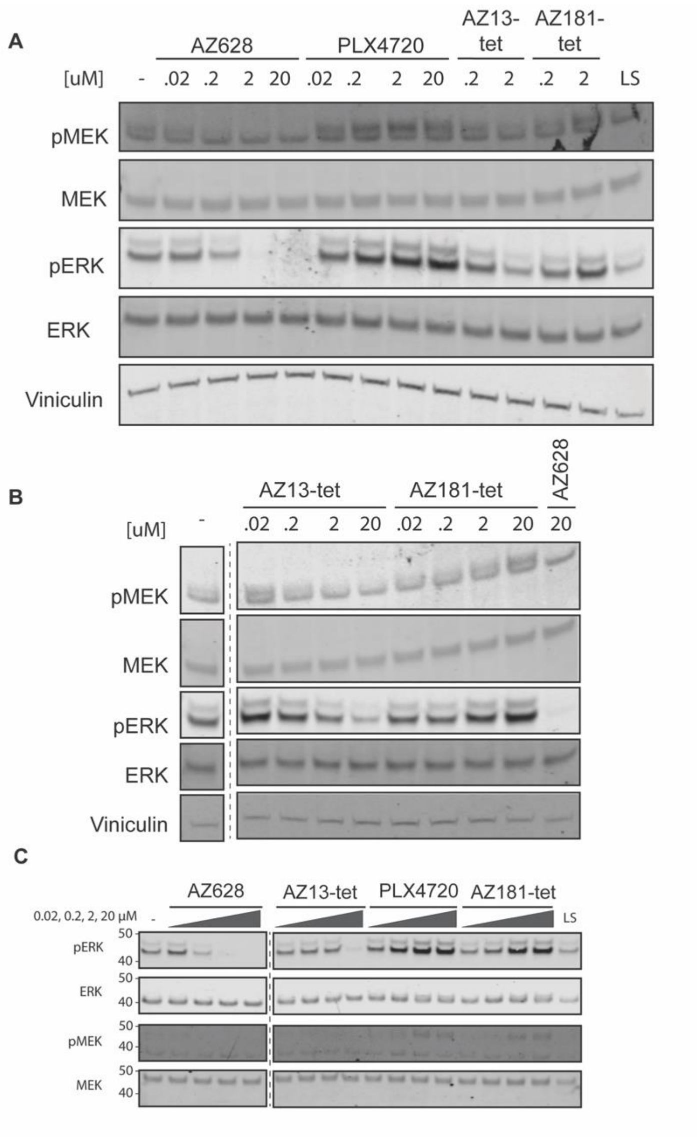

Figure S1. Cellular Response to Parental Pharmacophores and BOLT Ligands

A/B. The response of MAPK phosphorylation signaling was characterized using the indicated small molecules. HCT116 cells were treated with the indicated dose and small molecule for 2 hours and then lysed.

C. An independent experiment, with a focus on BOLT ligand response and incubation time of only 1 hour and comparison of lysates on a single blot. Lysates were immunoblotted with the indicated antibody. LS indicated low serum media conditions (0.1% FBS) for 12 hours. Dashed lines indicate omitted lanes in the blot.

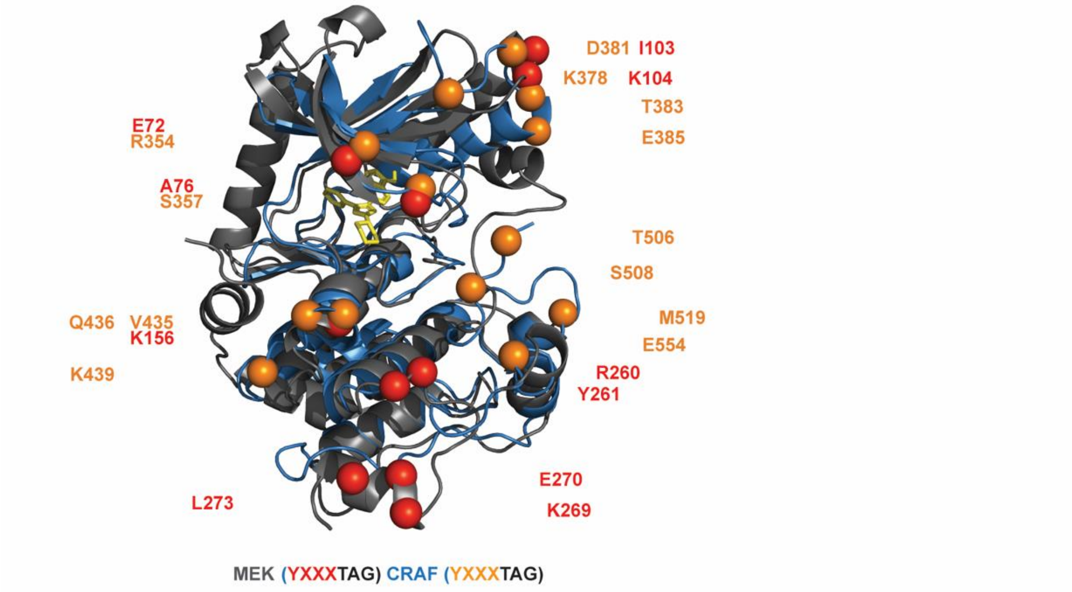

Figure S2. Kinase Domain Alignment of MEK and CRAF with ncAA Positions

Similar to main Figure 1D, structural superposition of MEK (grey) and CRAF (blue) kinase structures. A small molecule inhibitor (yellow) occupies the ATP binding pocket. Spheres highlight positions tested for amber suppression expression and tethering, MEK (red) CRAF (orange) YXXXTAG, where XXX indicates the position of the amber codon and Y indicates the canonical amino acid normally found at this position. Figure created using Pymol. PDB: 3ZLS MEK; 3OMV CRAF.

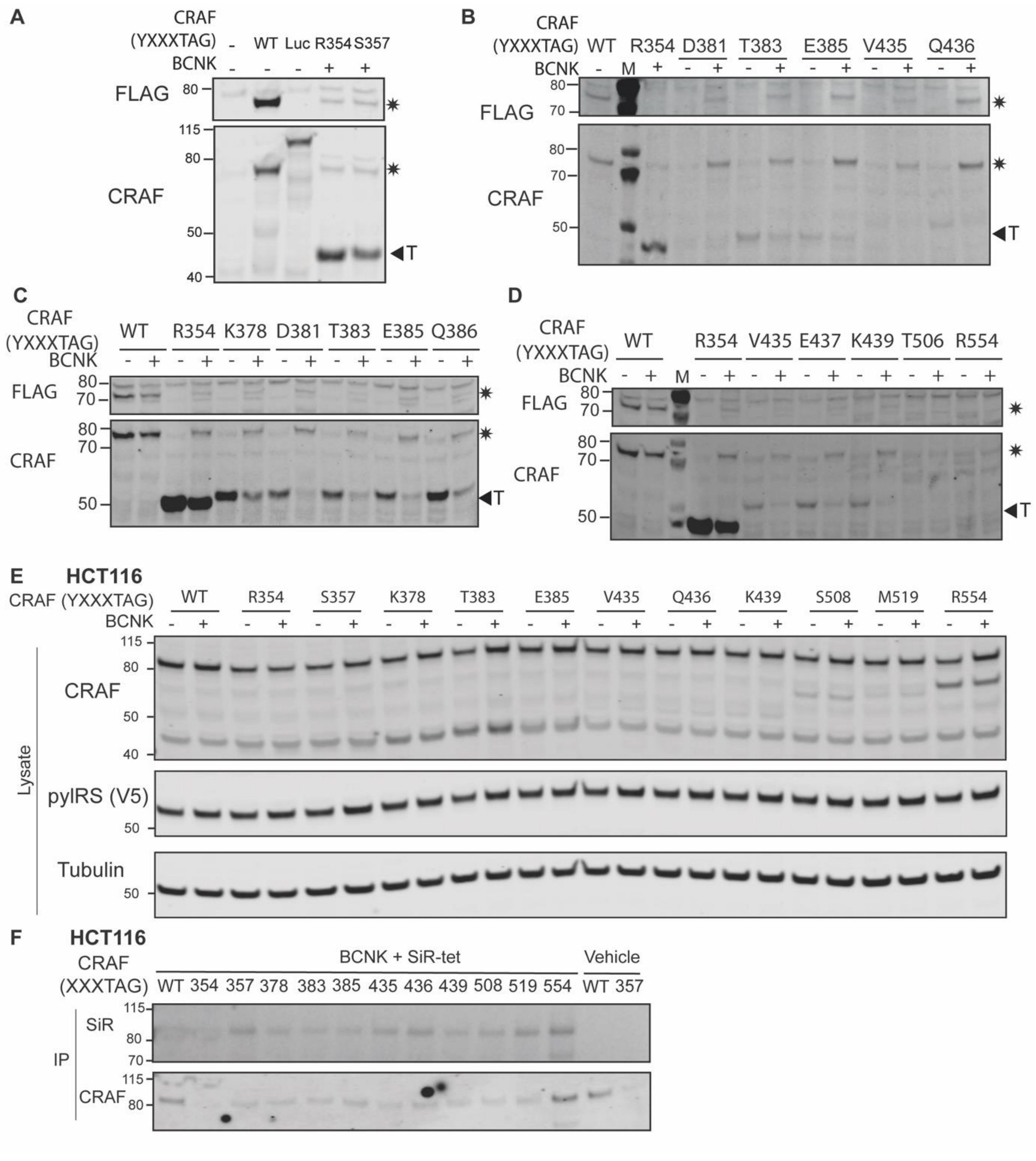

Figure S3. CRAF (YXXXTAG) and ncAA Dependent Expression

A-D. A HEK293 amber screening cell line, 293TReX-4xPylT-BFP-2A-*Mm*pylRS^{AF}, was transfected with the indicated CRAF-amber alleles. Transfected cells were grown in the presence of 200µM BCNK for a minimum of 24 hours. Lysates were analyzed by immunoblotting using FLAG-M2 (F1804 Sigma) and CRAF (9422 Cell Signaling Technologies). A lower molecular weight CRAF-amber protein product is indicated by a triangle (T). The approximate MW corresponds to translational termination at the amber codon. The star indicates expected full-length protein. We noted that the CRAF- antibody did not detect low

endogenous CRAF levels in HEK293 cells. In subsequent immunoblotting we utilized the BD CRAF antibody, which was more sensitive in our hands.

E. Indicated CRAF (YXXXTAG) alleles were co-transfected into HCT116 cells with plasmids expressing expanded genetic code machinery (*pylT* $PylRS^{AF}$). CRAF (YXXXTAG) alleles were expressed in the presence of 200 μM BCNK. Analysis of lysates was completed by immunoblotting. While most amber alleles show modest BCNK dependent increase in CRAF expression, the amber allele molecule weight is undistinguishable from endogenous CRAF. The expected FLAG epitope was not detected in bulk lysates in HCT116 screening. However, immunoprecipitation utilizing the FLAG epitope produced fluorescently labelled CRAF (YXXXTAG BCNK), see Figure S3F. Subsequent CRAF YXXXTAG variants employed the 3xFLAG-HA tag to facilitate immunoprecipitation and immunodetection. In addition, the dual epitope tag created a slight MW shift distinguished in SDS-PAGE, as seen in Figure 1E.

F. Immunoprecipitation and elution of CRAF (YXXXTAG) alleles from cell lysates, see E. Unincorporated ncAA was washed away prior to *in situ* labelling with Silicon Rhodamine-tetrazine (SiR-tet) dye. Resulting fluorescently labelled CRAF (YXXXTAG BCNK) is detected by *in gel* fluorescence.

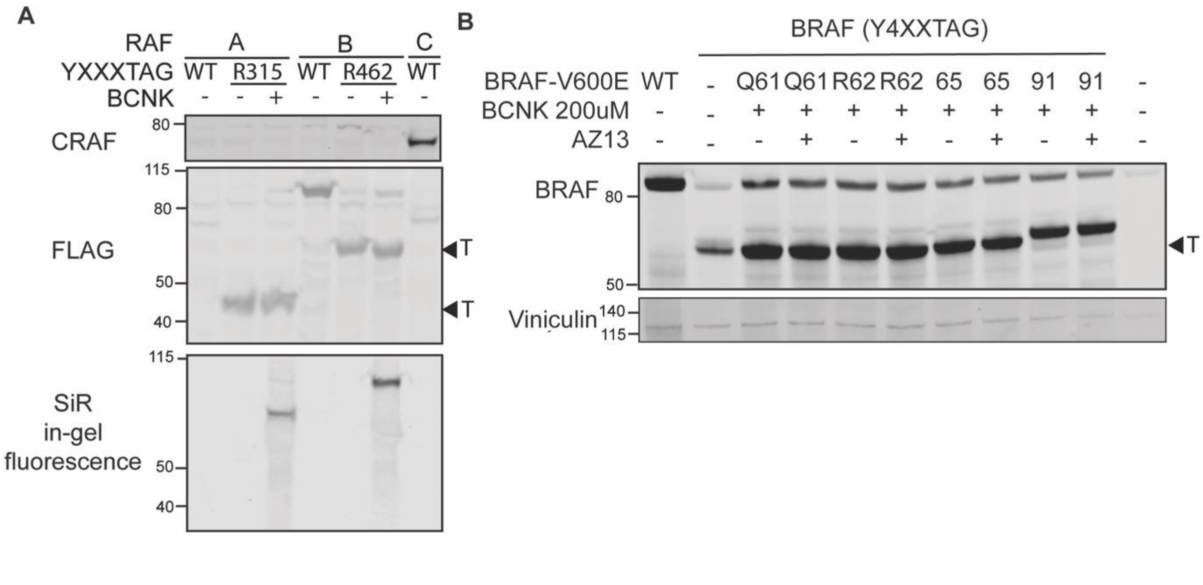

Figure S4. ARAF and BRAF (YXXXTAG) **Amber Allele Screen and ncAA Dependent Expression**

A. Alignments of the kinase domains of MEK and CRAF species were used to directed analogous amber variants in ARAF and BRAF species (N-terminal FLAG tagged). Positions in the N-lobe of the kinase domain were tested, proximal to residue R354 in CRAF, specifically ARAF R315 and BRAF R462. The HEK293 amber screening cell line, 293TReX-4x[*pylT]*-BFP-2A-*Mm*pylRS^{AF}, was transfected with the indicated RAF-amber alleles (A). Transfected cells were grown in the presence of 200uM BCNK for a minimum of 24 hours, washed, and then incubated with SiR-tet for *in situ* labelling. Lysates were collected and analyzed by *in gel* fluoresce and immunoblotting. Arrow heads indicated truncated products corresponding to the expected molecular weight of amber codon translational termination. Triangle T indicates truncated RAF species, resulting from lack of amber codon read through.

B. Screening and evaluation of the combined response of BRAF-V600E amber alleles positions with addition of AZ13-tet ligand. BRAF (YXXXTAG) alleles expression was strong on all tested amber sites, while substantial truncated protein product was detected, see triangle T.

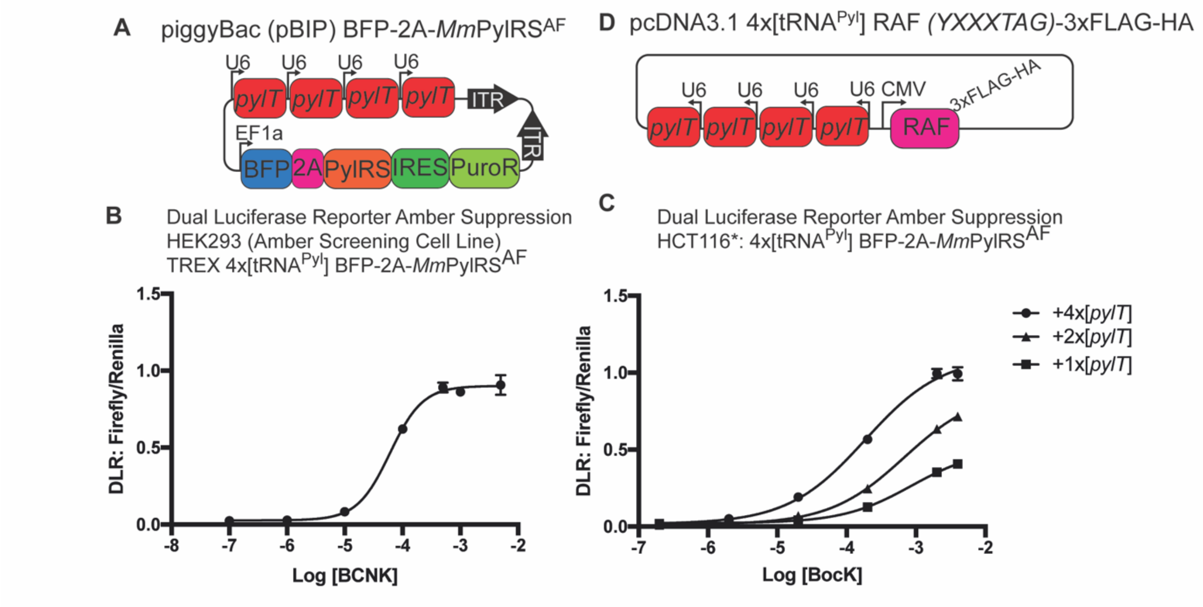

Figure S5. Evaluating Amber Suppression in HEK293 & HCT116* Stable Cell Lines

A. Schematic of PiggyBac vector for knock-in of genetic code expansion components, $tRNA^{Pyl}$ and amino-acyl synthetase PylRS. PylRS is co-expressed with blue fluorescent protein (BFP) separated by a ribosomal skipping 2A sequence. An IRES enables cistronic expression of Puromycin N-acetyl transferase (Puro)

B/C. dual luciferase reporter with the amber codon separating the independent luciferase sequences was transfected into HEK293 TReX 4x[*pylT]*-BFP-2A-*Mm*pylRS^{AF} and corresponding HCT116 BFP-2A-*Mm*pylRS^{AF} (HCT116*). The 293HEK showed a strong amber suppression ability, while the HCT116 cells showed a modest amber suppression response.

D. The additional transfection of *pylT* cassette enhanced the responsiveness of the HCT116* cells. As a result, a 4x [U6 promoter driving *pylT]* cassette was inserted into subsequent RAF (YXXXTAG) plasmids.

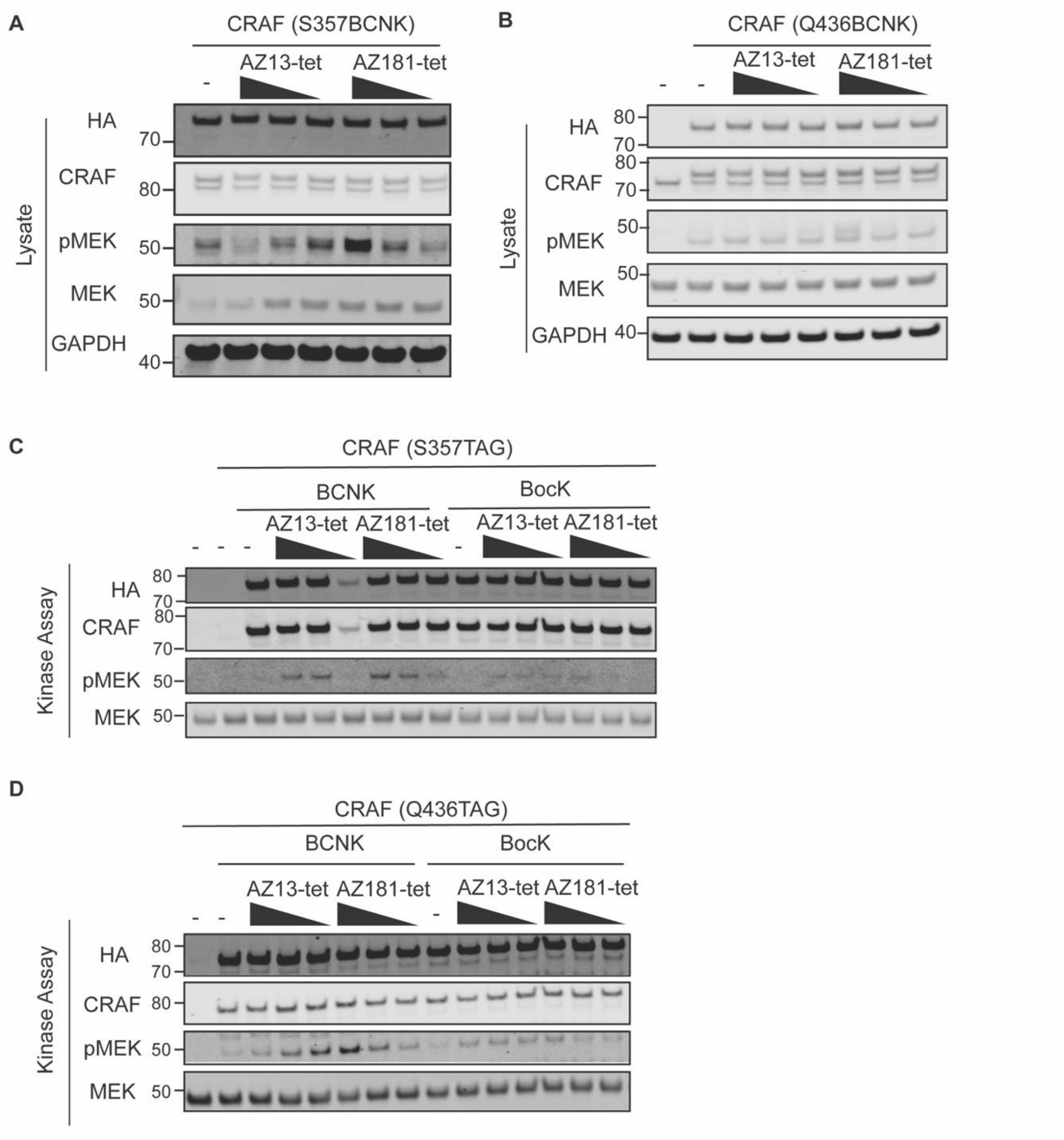

Figure S6. BOLT of CRAF (YXXXTAG) variants elicits kinase activation

A/B. Immunoblots corresponding to CRAF (YXXXTAG) BOLT experiments treated with indicated BOLT ligands for 2 hours (2000, 200 and 20 nM). V corresponds to vehicle DMSO control treatment. CRAF (YXXXTAG) expression runs at a higher molecular weight than endogenous CRAF as a result of the C-terminal 3xFLAG-HA epitope tag on amber alleles. As expected, paradoxical activation, i.e. increased pMEK, occurs with BOLT ligands, however it is difficult decipher the contribution of CRAF BOLT from parent pharmacophore action on cellular RAF species.

C/D. Original immunoblots that appear cropped in main Figure 2 C & D. Coimmunoprecipitation and kinase assay from CRAF (YXXXTAG) BOLT experiments.

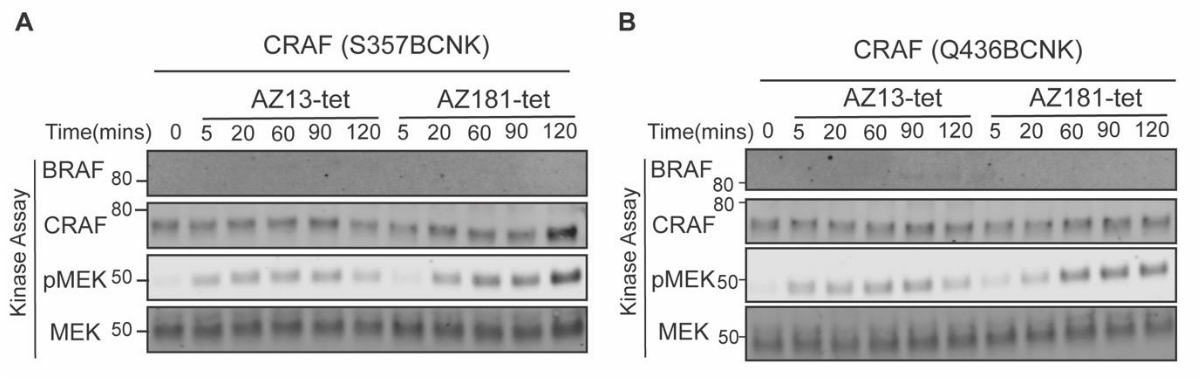

Figure S7. Kinetics of CRAF BOLT

A/B. Kinetics of CRAF BOLT mediated RAF transactivation. HCT116* cells were transfected with indicated CRAF (YXXXTAG) alleles and grown for 48 hours with 200 μM BCNK. The indicated BOLT ligand (2 μM) was incubated for the indicated time (minutes) and cells were then lysed. Resulting kinase activity was measured following FLAG immunoprecipitation and kinase assay described previously.

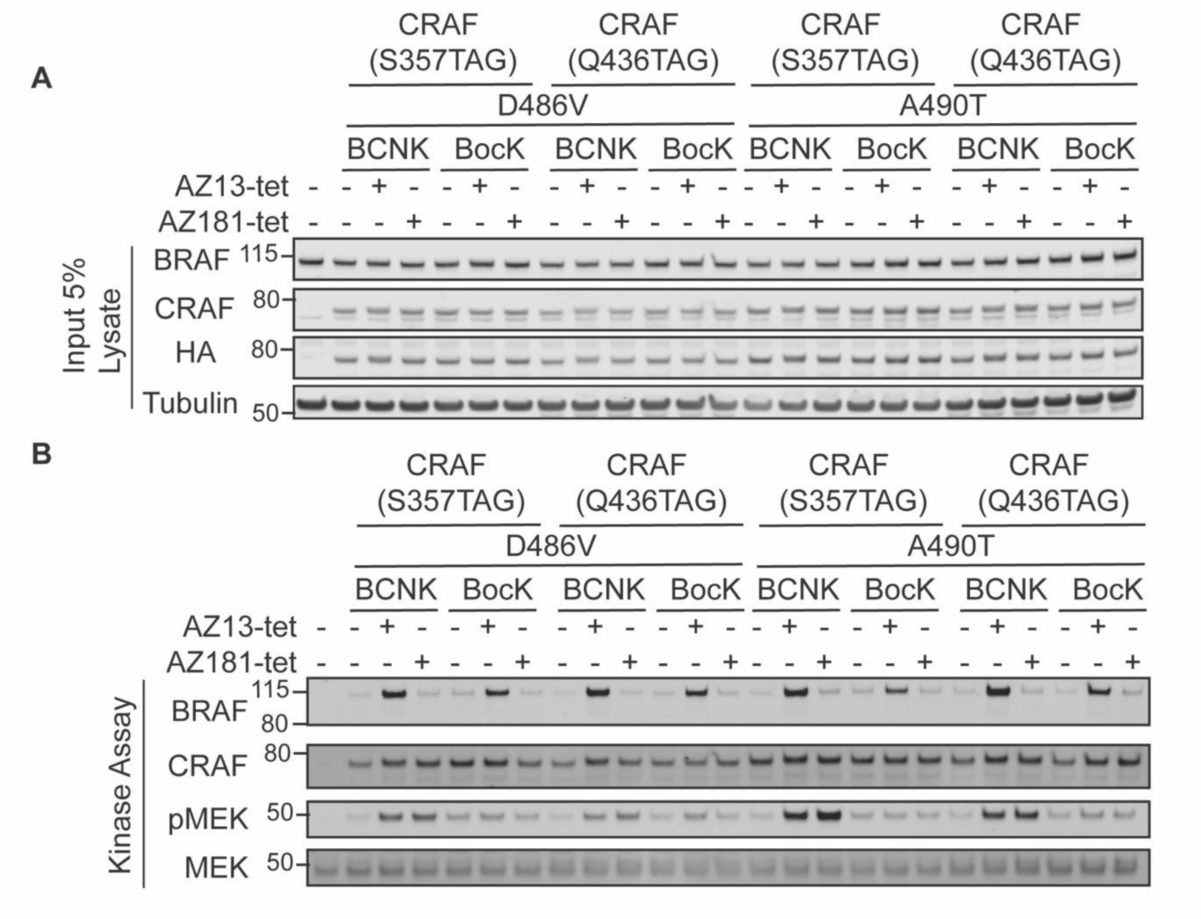

Figure S8. BOLT of kinase dead CRAF variants elicits kinase activation

A. Immunoblot of cell lysates co-expressing indicated kinase dead CRAF variants, CRAF(YXXTAG). HCT116* cells were co-transfected and incubated with ncAA (200 µM BCNK or 2 mM BocK). Cells were washed to remove excess ncAA and then incubated with vehicle, 1 uM AZ13-tet, or 1uM AZ181-tet for 2 hours.

B. Immunoblot of kinase assays form immunoprecipitated CRAF variants from panel A. As in Main Figure 2, the immunoprecipitate was assayed for RAF kinase activity *in vitro*, using catalytically dead MEK1 as a substrate. All CRAF amber alleles are HA tagged.

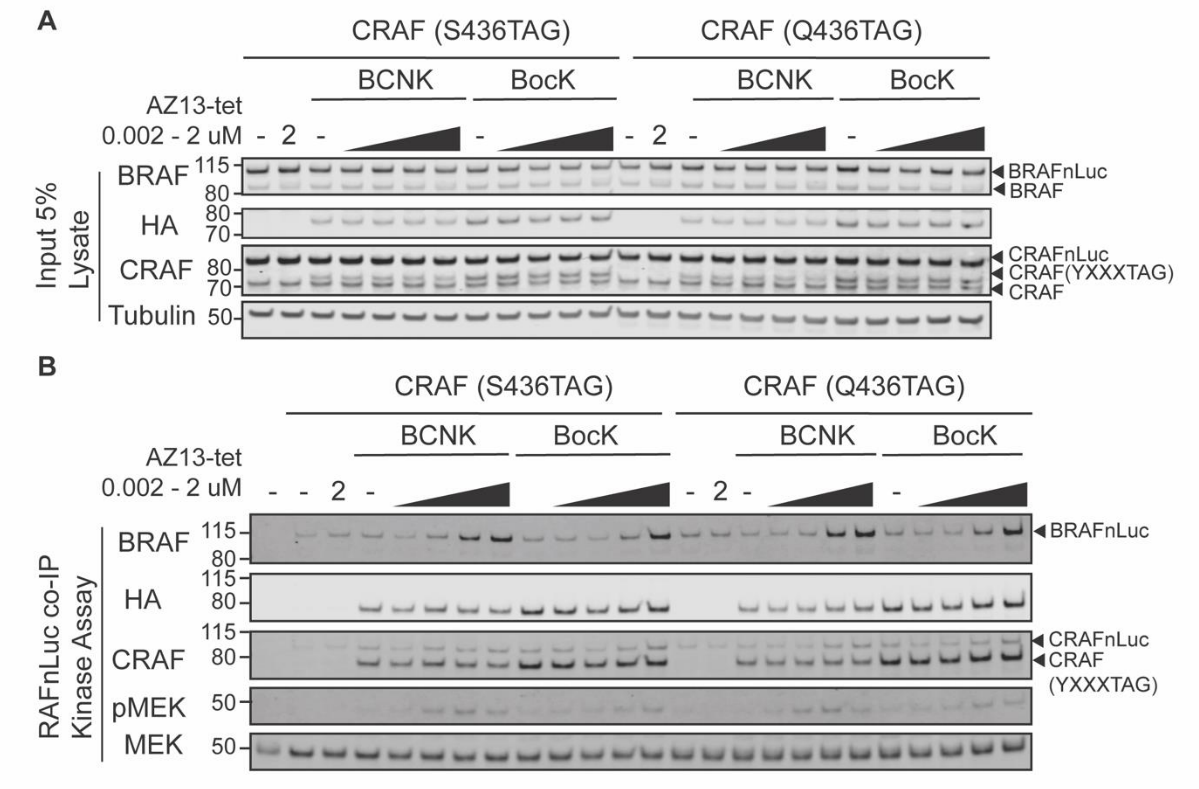

Figure S9. CRAF BOLT enhances co-immunoprecipitation of BRAF

A. Immunoblot of cell lysates co-expressing indicated RAF variants, CRAF(YXXTAG) and wildtype CRAF and BRAF fused to nanoluciferase (nLuc). HCT116* cells were co-transfected and incubated with ncAA (200 μM BCNK or 2 mM BocK). Cells were washed to remove excess ncAA and then incubated with increasing concentration of AZ13-tet (0, 2, 20, 200, 2000 nM) for 2 hours.

B. Co-immunoprecipitation of exogenous RAF species via C-terminal FLAG tag on CRAF(YXXTAG) variants, as expressed in panel A. The immunoprecipitate was assayed for RAF kinase activity in vitro, as described previously. All CRAF amber alleles are HA tagged.

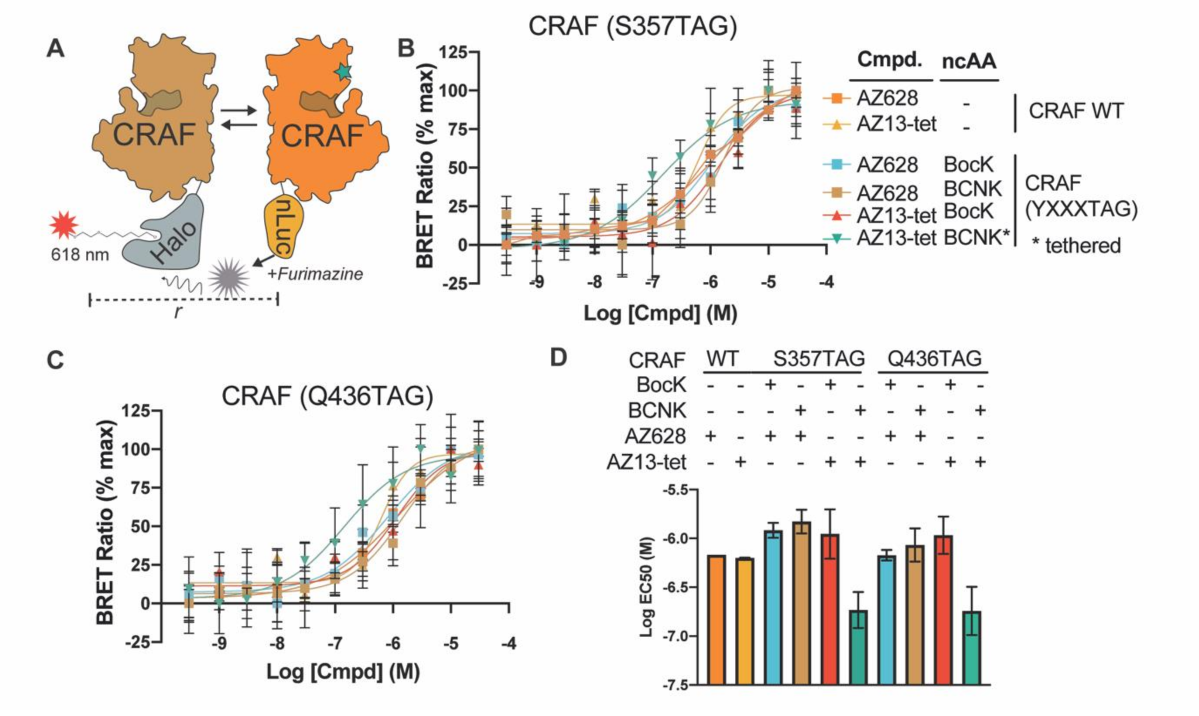

Figure S10. Homodimerization of cellular CRAF using BOLT

A. Cellular RAF dimerization assay based on the BRET donor nanoluciferase(nLuc) and BRET acceptor chloroalkane conjugate and halo tagged (HT) species of CRAF kinase. Addition of nLuc substrate, furimazine, results in a dimerization dependent energy transfer as detected by emission of the fluorescent-dye chloroalkane conjugate.

B. Homodimizeration of CRAFnLuc variants (wild-type and CRAF(S357TAG) incorporating BCNK or BocK) in response to increasing concentration of AZ628 and AZ13-tet. Error bars correspond to standard deviation across 4 biological replicates. Continuous line corresponds to non-linear regression (4 variable) completed using Prism software. Data are color coded as shown in the associated table.

C. Homodimizeration of CRAFnLuc variants (wild-type and CRAF(Q436TAG) incorporating BCNK or BocK) in response to increasing concentration of AZ628 and AZ13-tet. Error bars correspond to standard deviation across 4 biological replicates. Continuous line corresponds to non-linear regression (4 variable) completed using Prism software. Data are color coded as in panel **B**.

D. Summary of calculated apparent EC_{50} values across the different CRAF variants and ligands, mean values shown with error bars representing standard deviation between at least 2 independent dose response experiments. Error bars correspond to standard deviation between calculated EC_{50} values based on non-linear regression (4 variable) modelling.

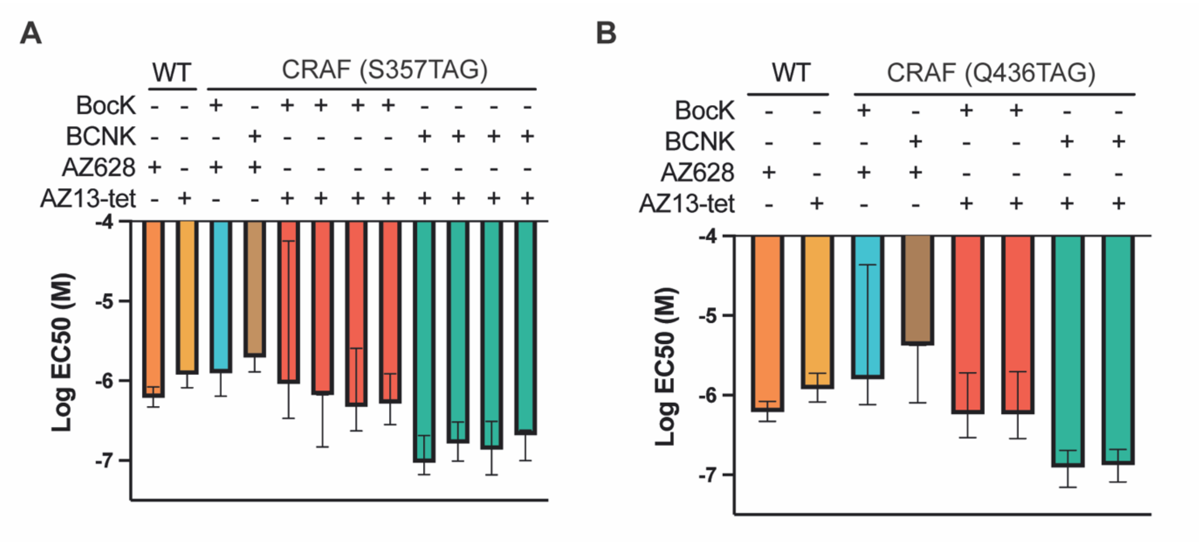

Figure S11. Asymmetric Confidence Intervals from nanoBRET Experiments

A/B. Calculated apparent EC_{50} values for heterodimerization, from independent dose response experiments for indicated CRAF alleles. The asymmetric confidence interval is a consequence of ligand solubility causing an under samples of doses for which nanoBRET ratios plateau in the assay. Each does response was completed over at least 8 concentrations, done in quadruplicate treatments. Following data processing, producing normalized response nanoBRET ratios, a four-parameter regression was calculated for each condition using graphpad. Error bars correspond to 95% confidence intervals from calculated regression.

MATERIALS AND METHODS:

Molecular Biology
Standard molecular biology techniques, including PCR, restriction enzyme cloning, Gibson assembly, quick change mutagenesis and site directed mutagenesis, were used to assemble the plasmids and constructs. Briefly, 3.1 4x[pylT] was made by Gibson assembly of the PCR product of 4x[U6-tRNAPyl] cassette and the backbone pCDNA3.1 digested with MfeI. The subsequent protein expression 3.14xpylT plasmids were assembled using a combination of restriction and Gibson assembly methods. Site directed mutagenesis was completed using quick change primers (Agilent primer design) or NEB base editor. As a result of the increased size of the backbone(~10kb), a split site in the Ampicillin resistance cassette was validated to enable more efficient PCR and Gibson assembly. Nanoluciferase fusions were inserted using PCR and Gibson assembly. Cloning for PiggyBac vectors (SBI) followed a similar approach as pcDNA3.1. Primers were ordered from IDT and Sigma, geneBlock fragments were ordered from IDT (Integrated DNA Technologies).

Biochemistry

Immunoprecipitations
Cells were washed in PBS, then lysed in NP40 lysis buffer (Life technologies) supplemented with protease and phosphatase inhibitors (ThermoFisher Scientific 78440). Clear lysates were added to anti-DYKDDDDK magnetic agarose beads (Pierce A36797) and gently mixed overnight at 4C. Beads were then washed with NP40 lysis buffer and washed twice with Kinase Activity Buffer (KAB), 30 mM Tris pH 7.4, 150 mM NaCl, 5mM $MgCl_2$, 0.5% NP40, 10% Glycerol)) at room temperature. Immunoprecipitated RAF complexes were eluted at room temperature using 150 ng/mL 3xFLAG peptide (Sigma) in KAB.

Kinase Assay
Eluted RAF complexes were added to KAB containing 50nM recombinant kinase-dead MEK1-K97M and 200 uM ATP, final concentration. Reactions was incubated at 30C for 30 minutes, quenched with the addition of loading buffer (4x LDS). Samples were then analysed by SDS-PAGE and immunoblotted with indicate antibodies.

Immunoblotting
Blots show a representative of at least three independent biological replicates. Following SDS-PAGE gel electrophoresis, samples were transferred to PVDF membranes using iBlot2 semi-dry transfer apparatus. Membranes were incubated with LICOR blocking buffer (PBS) for 30 min and then incubated overnight in primary antibody (diluted in 1:1 LICOR Blocking Buffer: PBS +%0.2. Tween-20). When appropriate multiple primary antibodies were used in parallel, otherwise duplicate gels and blotting was completed. Membranes were washed three times in PBS 0.1% Tween-20. Membranes were incubated with secondary LICOR antibodies for 45 min and washed three times in PBST before scanning using LICOR Odyssey scanner. Images were exported and analysed using ImageStudio (LICOR).

Antibodies:

BRAF (9433, Cell Signaling Technologies)
CRAF (610152, BD Sciences)

CRAF (53745, Cell Signaling Technologies)
ERK (4696, Cell Signaling Technologies)
pERK (4370, Cell Signaling Technologies)
FLAG-M2 (F1804, Sigma Aldrich)
GAPDH (2118, Cell Signaling Technologies)
HA-Rabbit (3724, Cell Signaling Technologies)
HA-Mouse (2367, Cell Signaling Technologies)
MEK1/2 (4694, Cell Signaling Technologies)
pMEK (9154, Cell Signaling Technologies)
Tubulin (3873, Cell Signaling Technologies)
Vinculin (13901, Cell Signaling Technologies)
V5-Rabbit (13202, Cell Signaling Technologies)
Goat anti-Mouse-680RD (925-68070, LICOR)
Goat anti-Rabbit-800CW (925-32211, LICOR)

Recombinant kinase dead MEK1 expression and purification
BL21 DE3 cells containing plasmid AZ-MEK1-KD-Avi-TEV-His6 was grown overnight to saturation and then used to inoculate a 1L flasks of 2XTY and grown at 37 C degrees until OD600 reached 0.8. MEK1 expression was induced with 300 uM IPTG and temperature was lowed to 18 C and allowed to express overnight (16+ hours). Cells were harvested by centrifugation, 4000g cells were lysed in in Lysis Buffer, 25 mM Tris pH 7.5, 500 mM NaCl, 10% glycerol, and 1 mM DTT and protease inhibitor cocktail (Roche). Cells were sonicated and supernatant collected following centrifugation 20000 g for 60 minutes. Supernatant was supplemented with 20 mM imidazole, pH 7.5 and loaded on a HisTrap column and washed with Buffer A, 25 mM Tris pH 7.5, 500 mM NaCl, 20 mM imidazole, 10% glycerol, and 1 mM DTT. Protein was eluted with Buffer A supplemented with 300mM imidazole. Fractions containing MEK1 were pulled, salt concentration was diluted, and then loaded onto an ion exchange column. Protein was eluted using a gradient mixing protocol of IEX A and B. IEX A 25 mM Tris pH 7.5, 5 % glycerol, 1 mM DTT and IEX B 25 mM Tris pH 7.5, 1 M NaCl, 5 % glycerol, 1 mM DTT.

RAF Dimerization NanoBRET
The manufacture's protocol was modified for the nBRET RAF assays. The RAF wildtype alleles were transiently transfected using Promega's pFC14K nLuc and HaloTag variants. RAF nLuc fusion are transfected at limiting concentrations to ensure robust assay responses. Wild type variants were transfected at a 10:1 ratio of HT:nLuc using HD FuGENE. Amber suppression of RAF (YXXXTAG) variants were negatively impacted by pFC14K backbone and final experiments utilized 3.1 4x[tRNAPyl]-CRAF(YXXXTAG)nLuc variants. Wildtype HaloTag variants remained in pFC14K backbone. nBRET RAF (YXXXTAG) variants were transfected at a 1:10 ratio of WT-HT: RAF(YXXXTAG)nLuc. Omission of conjugate-dye in adjacent wells allows for background fluorescent to be subtracted from BRET ratio signal. For dose response experiments, cells were transfected in parallel and ncAA added at the same time. Cells were grown for 48h and then split and re-plated into 384 well plates (40uL per well), with half of samples receiving HaloTag NanoBRET 618 ligand, in Opti-MEM without phenol red containing 4% FBS (8000-12000 cells per well). Cells were allowed to adhere for a minimum of 6 hours prior to dosing using a Tecan HP300 liquid dispenser. Compounds were incubated for 2 hours at 37C 10% CO2. Following incubation, nLuc substrate was prepared at 3X final concentration in 4% FBS OptiMEM and 20 uL was added to wells. Plates were immediately analysed on a

Pherastar FS using the NanoBRET filter module (610nm-LP, 460-80nm bandpass). Data was exported and analysed on Excel and graphed using Prism Graphpad. BRET ratio was normalized maximum signal response (% max) for each corresponding variant and ligand. Error bars indicate standard deviation across experimental replicates (n=4).

Cell Biology

Culturing & Transfections

HEK293 and HCT116 cells were cultured in DMEM or McCoy's 5A media, respectively, media was supplemented with 10% FBS and penicillin and streptomycin, 37C 5% CO2 atmosphere. Cells were transiently transfected with HD FuGENE (Promega) according to the manufacturer's protocol. Following overnight incubation with transfection complex, cells were split and the corresponding ncAA was supplemented to the growth media. Liquid stocks of ncAA (BCNK and BocK) were made by dissolving powdered ncAA in 200 mM NaOH and sonicating. Transfections for nBRET experiments were completed in replicates with ncAA being added at the time of transfection to maximize amber allele expression. For immunoblotting cells were washed with PBS and lysed with RIPA lysis buffer, supplemented with protease inhibitors (Halt Protease & Phosphatase Inhibitor Cocktail, Pierce). Lysates were stored at -20C until analysis by SDS-PAGE and immunoblotting. See antibody list for primary and LICOR secondary antibodies.

Stable Cell Generation

Cell lines were made using transposon mediated integration of indicated cassettes. First a PiggyBac (pB) vector encoding 4x[tRNAPyl]-BFP-2A-MmPylRS(Y306A, Y384F) (AF) was knocked-in using co-expression of the PiggyBac transposase (SBI) selection was achieved with 1 ug/mL puromycin. Cells surviving selection were sorted by FACS with gating on BFP+ cells. These parental amber suppression cells were used for transient transfection of CRAF(YXXXTAG) alleles for immunoprecipitation, kinase assays.

Ligand Design & Tethering Compatibility

The residues explored as sites for incorporation of the ncAA were based on the homologous residues to those explored in the original MEK BOLT constructs. The prioritization of these residues and the design of BOLT probes were based on protein-inhibitor crystal structures of the "parent" ligands AZ628 and PLX4720 bound to BRAF kinase*. The two residues focused on for the incorporation of ncAA (CRAF S357 and Q436) are located on two domains of the CRAF kinase structure adjacent to the solvent channel of the ATP (inhibitor) binding cleft and proximal to the location of the attachment points of the linker to the RAF ligands. The linker length between RAF binding ligand and tetrazine covalent tethering group was empirically selected from simple modelling. The linear through space distance from the α-carbon of the equivalent ncAA residues (BRAF: S465 & I544 ; CRAF S357 & Q436) for BOLT attachment residues and the linker attachment point on AZ628 is 13-14 Å (cis binding) while the attachment point on AZ628 in the other protomer (trans binding) is 36-39 Å.

* The BRAF inhibitor crystal structures used in the design work were AZ in house generated structures but these are nearly identical to publicly deposited structures for AZ-628 (PDB code 4RZW) and PLX4720 (PDB code 3C4C).

SYNTHETIC PROCEDURES

RAF BOLT Ligand Synthesis

tert-Butyl N-l[(4-cyanophenyl)methyl]carbamate

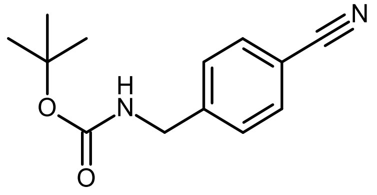

A solution of 4-cyanobenzylamine hydrochloride (10 g, 59.30 mmol) in water (50 mL) was added to a stirred solution of NaOH (7.12 g, 177.91 mmol) and di-tert-butyl dicarbonate (15.15 mL, 65.23 mmol) in water (50 mL) at 20°C. The resulting mixture was stirred at 25 °C for 16 hours. The precipitate was collected by filtration, washed with water (150 mL) and air dried to afford tert-butyl N-l[(4-cyanophenyl)methyl]carbamate (12.50 g, 91 %) as a white solid, which was used without further purification. 1H NMR (400MHz, CDCl3) δ 1.48 (9H, s), 4.39 (2H, t), 4.99 (1H, br), 7.40 (2H, d), 7.63-7.65 (2H, m); m/z (ES+), [M+H]+ = 233

tert-Butyl N-[[4-(1,2-dihydro-1,2,4,5-tetrazin-3-yl)phenyl]methyl]carbamate

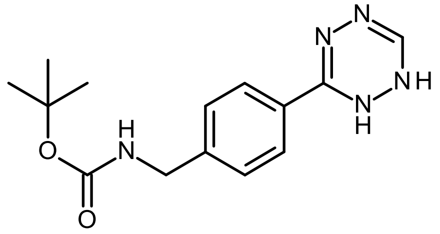

Hydrazine (8.61 mL, 219.56 mmol) was added in one portion to tert-butyl-N-l[(4-cyanophenyl)methyl]carbamate (1g, 4.31 mmol), formamidine acetate (4.48 g, 43.05 mmol) and zinc trifluoromethanesulfonate (0.782 g, 2.15 mmol) in at rt. The resulting mixture was stirred at 30 °C for 36 hours. The mixture was diluted with EtOAc (100 mL), and the organic phase was washed with water, brine and dried over Na2SO4, filtered and evaporated to afford tert-butyl N-[[4-(1,2-dihydro-1,2,4,5-tetrazin-3-yl)phenyl]methyl]carbamate
(1.100 g, 88 %) as a yellow oil which was used without further purification.
m/z (ES+), [M+H]+ = 290

tert-Butyl N-[[4-(1,2,4,5-tetrazin-3-yl)phenyl]methyl]carbamate

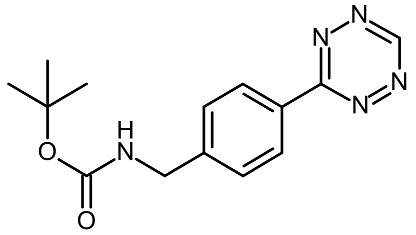

Sodium nitrite (11.92 g, 172.81 mmol) was added portionwise to tert-butyl N-[[4-(1,2-dihydro-1,2,4,5-tetrazin-3-yl)phenyl]methyl]carbamate (2.5 g, 8.64 mmol) in DCM/AcOH (1:1) (120 mL) at rt and the resulting mixture was stirred at 25 °C for 0.5 hour. The mixture was diluted with DCM (100 mL), and the organic phase was washed with water (50 mL), NaHCO3 (aq.) and dried over Na2SO4, filtered and evaporated to give crude product. The crude product was purified by flash silica chromatography, elution gradient 10 to 30% EtOAc in petroleum ether. Pure fractions were evaporated to dryness to afford tert-butyl N-[[4-(1,2,4,5-tetrazin-3-yl)phenyl]methyl]carbamate (1.500 g, 60.4 %) as a pink solid. 1H NMR (400 MHz, DMSO-d6) δ 1.42 (9H, s), 4.27 (2H, d), 7.54 (3H, d), 8.47 (2H, d), 10.58 (1H, s); m/z (ES+), [M-tBu]+ = 232

[4-(1,2,4,5-Tetrazin-3-yl)phenyl]methanamine

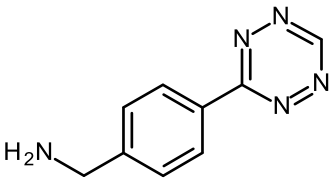

HCl (4M in dioxane) (40 ml, 1316.6 mmol) was added to tert-butyl N-[[4-(1,2,4,5-tetrazin-3-yl)phenyl]methyl]carbamate (1.4 g, 4.87 mmol) in DCM (80 mL). The resulting solution was stirred at 25 °C for 1 hour. The solvent was removed under reduced pressure to afford [4-(1,2,4,5-tetrazin-3-yl)phenyl]methanamine hydrochloride (0.500 g, 54.2 %) as pink solid. 1H NMR (400 MHz, DMSO-d6) δ 4.18 (2H, q), 7.77 - 7.84 (2H, m), 8.50 - 8.58 (2H, m), 8.65 (3H, s), 10.64 (1H, s); m/z (ES+), [M+H]+ = 188

2,6-difluoro-3-nitro-benzoylchloride

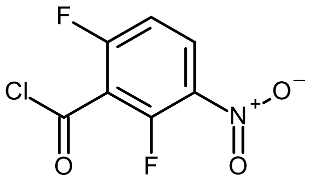

Oxalyl dichloride (12.58 mL, 147.71 mmol) was added portionwise to DMF (0.572 mL, 7.39 mmol) and 2,6-difluoro-3-nitro-benzoic acid (15 g, 73.85 mmol) in DCM (300 mL) at 10°C. The resulting solution was stirred at rt for 16 hours. The reaction mixture was evaporated to afford 2,6-difluoro-3-nitro-benzoyl chloride (17.30 g, 106 %) as a black oil, which was used without further purification. 1H NMR (300 MHz, CD3OD) δ 7.20-7.25 (1H,m), 8.40-8.48 (1H,m); m/z (ES+), [M+H]+ = 222

(5-Bromo-1H-pyrrolo[2,3-b]pyridin-3-yl)-(2,6-difluoro-3-nitro-phenyl)methanone

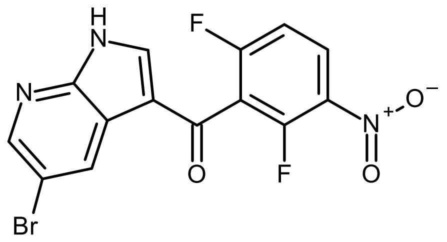

2,6-difluoro-3-nitro-benzoyl chloride (17.3 g, 78.09 mmol) was added portionwise to aluminum chloride (41.6 g, 312.35 mmol) and 5-bromo-1H-pyrrolo[2,3-b]pyridine (15.40 g, 78.17 mmol) in DCE (100 mL) at 20 °C under nitrogen. The resulting mixture was stirred at 50 °C for 17 hours. The reaction mixture was quenched with a 1:1 mixture of water and CH3CN (100 mL). The precipitate was collected by filtration, washed with water (100 mL), CH3CN (200 mL) and dried in the vacuum oven to afford (5-bromo-1H-pyrrolo[2,3-b]pyridin-3-yl)-(2,6-difluoro-3-nitro-phenyl)methanone (14.80 g, 49.6 %) as a brown solid, which was used without further purification. m/z (ES+), [M+H]+ = 382

(3-Amino-2,6-difluoro-phenyl)-(5-bromo-1H-pyrrolo[2,3-b]pyridin-3-yl)methanone

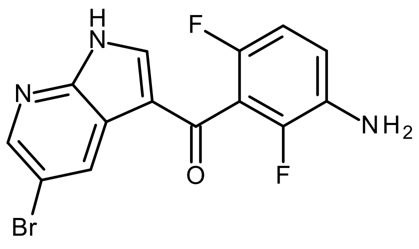

Tin(II) chloride (29.4 g, 154.92 mmol) was added to (5-bromo-1H-pyrrolo[2,3-b]pyridin-3-yl)-(2,6-difluoro-3-nitro-phenyl)methanone (14.8 g, 38.73 mmol) in THF (100 mL) at 20 °C . The resulting solution was stirred at 60 °C for 9 hours. The reaction mixture was quenched with Sat. potassium carbonate solution (25 mL). The solids were removed by filtration and washed with THF. The organic phase was washed with brine. The organic layer was dried over Na2SO4, filtered and evaporated to afford crude (3-amino-2,6-difluoro-phenyl)-(5-bromo-1H-pyrrolo[2,3-b]pyridin-3-yl)methanone (15.10 g, 111 %) as

a brown solid, which was used without further purification. 1H NMR (400 MHz, DMSO-d6) δ 5.23 (2H, s), 6.82 - 7.00 (2H, m), 8.09 - 8.19 (1H, m), 8.49 (1H, d), 8.52 - 8.63 (1H, m), 13.06 (1H, d); m/z (ES+), [M+H]+ = 352

N-[3-(5-Bromo-1H-pyrrolo[2,3-b]pyridine-3-carbonyl)-2,4-difluoro-phenyl)propane-1-sulfonamide

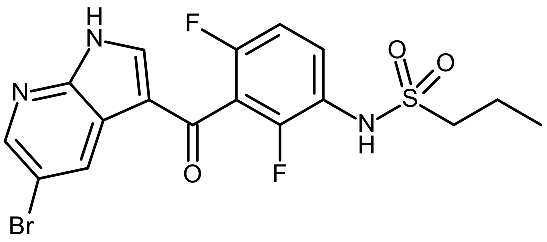

Propane-1-sulfonyl chloride (9.11 g, 63.90 mmol) was added to (3-amino-2,6-difluoro-phenyl)-(5-bromo-1H-pyrrolo[2,3-b]pyridin-3-yl)methanone (15 g, 42.60 mmol) and pyridine (6.89 mL, 85.19 mmol) in DCM (200 mL) at 15°C . The resulting mixture was stirred at rt for 16 hours. The mixture was filtered through a Celite pad. The filtrate was quenched with water (400 mL), extracted with DCM (3 x 200 mL), the organic layer was dried over Na_2SO_4, filtered and evaporated to afford N-[3-(5-bromo-1H-pyrrolo[2,3-b]pyridine-3-carbonyl)-2,4-difluoro-phenyl)propane-1-sulfonamide (8.60 g, 44.1 %) as yellow oil. This was used without further purification. m/z (ES+), [M+H]+ = 458

N-[3-[5-Bromo-1-(2-(trimethylsilylethoxymethyl)pyrrolo[2,3-b]pyridine-3-carbonyl]-2,4-difluoro-phenyl]-N-(2-trimethylsilylethoxy)methyl)propane-1-sulfonamide

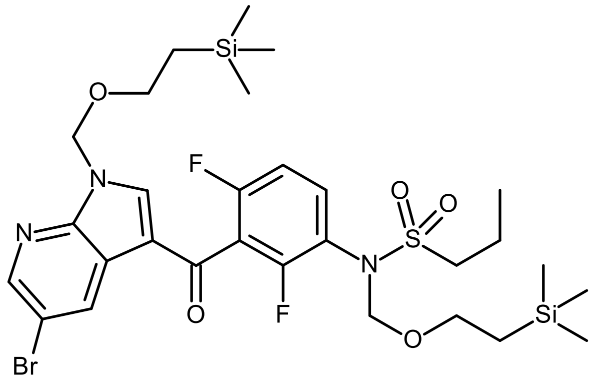

(2-(chloromethoxy)ethyl)trimethylsilane (5.89 g, 35.35 mmol) was added dropwise to N-[3-(5-bromo-1H-pyrrolo[2,3-b]pyridine-3-carbonyl)-2,4-difluoro-phenyl)propane-1-sulfonamide (5.4 g, 11.78 mmol) and Cs_2CO_3 (15.36 g, 47.13 mmol) in DMF (150 mL) at 0 °C. The resulting mixture was stirred at 25 °C for 18 hours. The mixture was filtered through a Celite pad and the filtrate was diluted with EtOAc (500 mL) and the organic

phase was washed with water, brine then dried over Na2SO4, filtered and evaporated to afford crude product. The crude product was purified by flash silica chromatography, elution gradient 0 to 25% EtOAc in petroleum ether. Pure fractions were evaporated to dryness to afford N-[3-[5-bromo-1-(2-(trimethylsilylethoxymethyl)pyrrolo[2,3-b]pyridine-3-carbonyl]-2,4-difluoro-phenyl]-N-(2-trimethylsilylethoxy)methyl)propane-1-sulfonamide (5.90 g, 69.7 %) as a yellow oil. This was used without further purification. m/z (ES+), [M+H]+ = 718

N-[2,4-Difluoro-3-[5-hydroxy-1-(2-(trimethylsilylethoxymethyl)pyrrolo[2,3-b]pyridine-3-carbonyl]phenyl]-N-(2-(trimethylsilylethoxymethyl)propane-1-sulfonamide

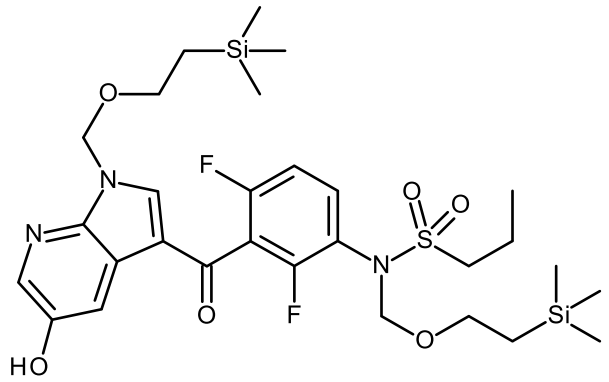

KOH (1M solution in water) (6.68 mL, 6.68 mmol) was added in one portion to N-[3-[5-bromo-1-(2-(trimethylsilylethoxymethyl)pyrrolo[2,3-b]pyridine-3-carbonyl]-2,4-difluoro-phenyl]-N-(2-trimethylsilylethoxy)methyl)propane-1-sulfonamide (1.2g, 1.67 mmol) in dioxane (60mL) at rt under nitrogen. The resulting mixture was stirred at 95 °C for 6 hours. The reaction was diluted with EtOAc (100 mL). The organic phase was washed with water and brine then dried over Na2SO4, filtered and the solvent evaporated. The resulting crude product was purified by flash silica chromatography, elution gradient 0 to 20% EtOAc in petroleum ether. Pure fractions were evaporated to dryness to afford N-[2,4-difluoro-3-[5-hydroxy-1-(2-(trimethylsilylethoxymethyl)pyrrolo[2,3-b]pyridine-3-carbonyl]phenyl]-N-(2-(trimethylsilylethoxymethyl)propane-1-sulfonamide (0.214 g, 19.54 %) as a brown oil. m/z (ES+), [M+H]+ = 656

Ethyl 6-[3-[2,6-difluoro-3-[propylsulfonyl(2-trimethylsilylethoxymethyl)amino]benzoyl]-1-(2-trimethylsilylethoxymethyl)pyrrolo[2,3-b]pyridin-5-yl]oxyhexanoate

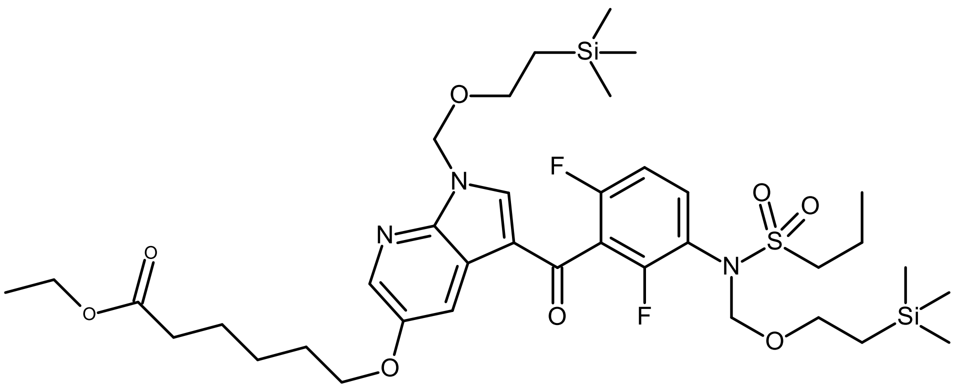

Ethyl 6-bromohexanoate (102 mg, 0.46 mmol) was added in one portion to N-[2,4-difluoro-3-[5-hydroxy-1-(2-(trimethylsilylethoxymethyl)pyrrolo[2,3-b]pyridine-3-carbonyl]phenyl]-N-(2-(trimethylsilylethoxymethyl)propane-1-sulfonamide (214 mg, 0.33 mmol), and Cs2CO3 (213 mg, 0.65 mmol) in DMF (5 mL) at rt. The resulting mixture was stirred at rt for 12 hours. The mixture was diluted with EtOAc (100 mL), and the organic phase was washed with water, brine and the organic phase was dried over Na2SO4, filtered and evaporated to give ethyl 6-[3-[2,6-difluoro-3-[propylsulfonyl(2-trimethylsilylethoxymethyl)amino]benzoyl]-1-(2-trimethylsilylethoxymethyl)pyrrolo[2,3-b]pyridin-5-yl]oxyhexanoate (215 mg, 83 %) as brown oil, which was used without further purification. m/z (ES+), [M+H]+ = 798

Methyl 6-[[3-[2,6-difluoro-3-(propylsulfonamido)benzoyl]-1H-pyrrolo[2,3-b]pyridin-5-yl]oxy]hexanoate

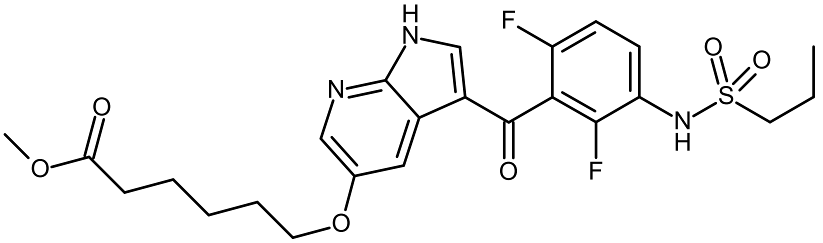

2M Hydrochloric acid (10 mL, 20.00 mmol) was added in one portion to ethyl 6-[3-[2,6-difluoro-3-[propylsulfonyl(2-trimethylsilylethoxymethyl)amino]benzoyl]-1-(2-trimethylsilylethoxymethyl)pyrrolo[2,3-b]pyridin-5-yl]oxyhexanoate (215 mg, 0.27 mmol) in MeOH (10 mL) at rt. The resulting mixture was stirred at 60 °C for 16 hours. The reaction mixture was poured into saturated K2CO3 (100 mL), extracted with EtOAc (2 x 50 mL), the organic layer was dried over Na2SO4, filtered and evaporated to afford methyl 6-[[3-[2,6-difluoro-3-(propylsulfonamido)benzoyl]-1H-pyrrolo[2,3-b]pyridin-5-yl]oxy]hexanoate (143 mg, 100 %) as yellow oil, which was used without further purification. m/z (ES+), [M+H]+ = 524.

6-[[3-[2,6-Difluoro-3-(propylsulfonamido)benzoyl]-1H-pyrrolo[2,3-b]pyridin-5-yl]oxy]hexanoic acid

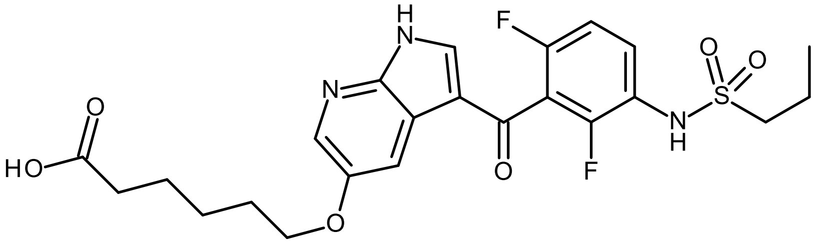

LiOH (1.366 mL of 2M solution in water, 2.73 mmol) was added in one portion to methyl 6-[[3-[2,6-difluoro-3-(propylsulfonamido)benzoyl]-1H-pyrrolo[2,3-b]pyridin-5-yl]oxy]hexanoate (143 mg, 0.27 mmol) in MeOH (10 mL) at rt. The resulting mixture was stirred at rt for 16 hours. The mixture was evaporated to give crude, which was diluted with water (10 mL) and the aqueous phase was acidified with 1N hydrochloric acid. The resulting solid was collected by filtration to afford 6-[[3-[2,6-difluoro-3-(propylsulfonamido)benzoyl]-1H-pyrrolo[2,3-b]pyridin-5-yl]oxy]hexanoic acid (64.0 mg, 46.0 %) as brown solid, which was used without further purification. m/z (ES+), [M+H]+ = 510

6-[[3-[2,6-Difluoro-3-(propylsulfonylamino)benzoyl)-1H-pyrrolo[2,3-b]pyridin-5-yl]oxy-N-[[4-(1,2,4,5-tetrazin-3-yl)phenyl]methyl]hexanamide (AZ181-tet)

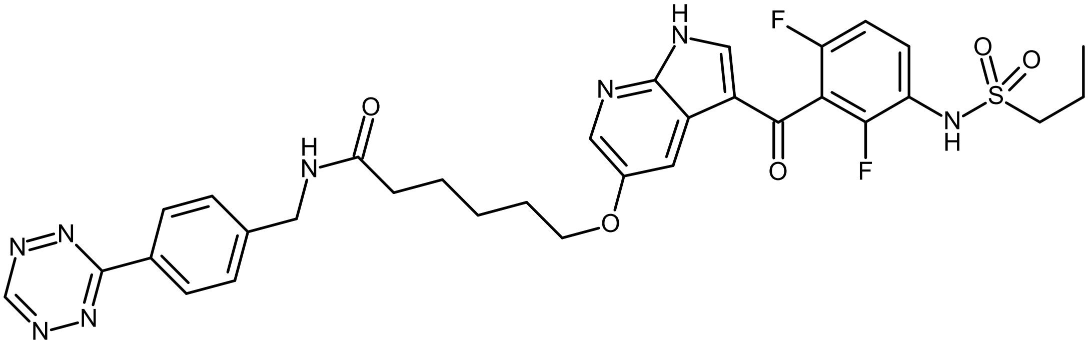

(Benzotriazol-1-yloxy)tripyrrolidinophosphonium hexafluorophosphate (15.32 mg, 0.03 mmol) was added to 6-[[3-[2,6-difluoro-3-(propylsulfonamido)benzoyl]-1H-pyrrolo[2,3-b]pyridin-5-yl]oxy]hexanoic acid (10.0 mg, 0.02 mmol), [4-(1,2,4,5-tetrazin-3-yl)phenyl]methanamine hydrochloride (6.58 mg, 0.03 mmol) and DIPEA (0.021 mL, 0.12 mmol) in DCM (2 mL) at rt . The resulting solution was stirred at 25 °C for 16 hours, and then the mixture was diluted with DCM (100 mL). The organic phase was washed with brine then dried over Na2SO4, filtered and evaporated. The resulting crude product

was purified by flash silica chromatography, elution gradient 0 to 5% MeOH in DCM. The semi-purified product was purified by preparative HPLC. Column: XSelect CSH Prep C18 OBD Column, 19x250mm,5um; Mobile Phase A:Water(0.1%FA), Mobile Phase B: ACN; Flow rate: 25 mL/min; Gradient: 45% B to 63% B in 7 min; 254/220 nm; Rt: 5.97 min Fractions containing the desired compound were evaporated to dryness to afford 6-[[3-[2,6-difluoro-3-(propylsulfonylamino)benzoyl)-1H-pyrrolo[2,3-b]pyridin-5-yl]oxy-N-[[4-(1,2,4,5-tetrazin-3-yl)phenyl]methyl]hexanamide (AZ181-tet) (9.40 mg, 69 %) as a red solid. 1H NMR (400 MHz, DMSO-d6) δ 0.96 (3H, t, J = 7.4 Hz), 1.49 (2H, q, J = 8.5 Hz), 1.66 (2H, dd, J = 14.7, 7.2 Hz), 1.7 – 1.85 (4H, m), 2.24 (2H, t, J = 7.4 Hz), 3.07 – 3.15 (2H, m), 4.08 (2H, t, J = 6.4 Hz), 4.41 (2H, d, J = 5.9 Hz), 7.25 (1H, t, J = 8.8 Hz), 7.55 (2H, d, J = 8.3 Hz), 7.56 – 7.61 (1H, m), 7.95 (1H, s), 8.06 (1H, s), 8.12 (1H, d, J = 2.8 Hz), 8.45 (2H, d, J = 8.3 Hz), 8.47 – 8.52 (1H, m), 9.75 (1H, s), 10.56 (1H, s), 12.76 (1H, s); 13C NMR (126 MHz, DMSO-d6) 12.68 (CH3), 16.95 (CH2), 25.07 (CH2), 25.21 (CH2), 28.46 (CH2), 35.32 (CH2), 41.84 (CH2), 53.43 (CH2), 68.43 (CH2), 112.05 (CH), 115.27 (C), 117.69 (C), 127.81 (CH), 128.10 (CH), 130.33 (C), 135.69 (CH), 144.14 (C), 145.08 (C), 152.33 (C), 158.07 (CH), 165.42 (C), 172.33 (C), 180.72 (C); 19F NMR (400 MHz, DMSO) δ -122.6; m/z (ES+), [M+H]+ = 679; HRMS: Calculated for $C_{32}H_{32}N_8O_5F_2S$ 679.2263, found 679.2255, error 1.2 ppm

Ethyl 5-(6-bromo-4-oxo-quinazolin-3-yl)pentanoate

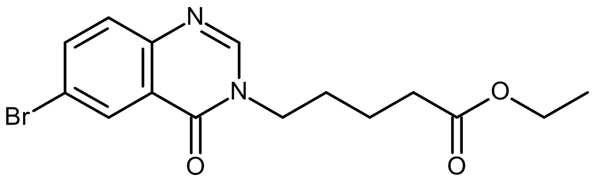

Ethyl 5-bromopentanoate (4.83 g, 23.11 mmol) was added to 6-bromo-3H- quinazolin-4-one (4 g, 17.77 mmol) and K2CO3 (6.39 g, 46.21 mmol) in DMF (5 mL) at 20°C. The resulting mixture was stirred at 70 °C for 16 hours and then poured into ice water. The precipitate was collected by filtration, washed with water and dried under vacuum to afford crude product. The crude product was purified by flash silica chromatography, elution gradient 10 to 40% EtOAc in petroleum ether. Pure fractions were evaporated to dryness to afford ethyl 5-(6-bromo-4-oxo-quinazolin-3-yl)pentanoate (5.20 g, 83 %) as a white solid. 1H NMR (400MHz, CDCl3) δ 1.26 (3H, t), 1.67-1.75 (2H, m), 1.81-1.88 (2H, m), 2.37 (2H, t), 4.02 (2H, t), 4.09-4.16 (2H, m), 7.56 (1H, d), 7.81-7.83 (1H, m), 8.04 (1H, s), 8.42 (1H, d); m/z (ES+), [M+H]+ = 353

Ethyl 5-[6-(2-methyl-5-nitro-anilino)-4-oxo-quinazolin-3-yl]pentanoate

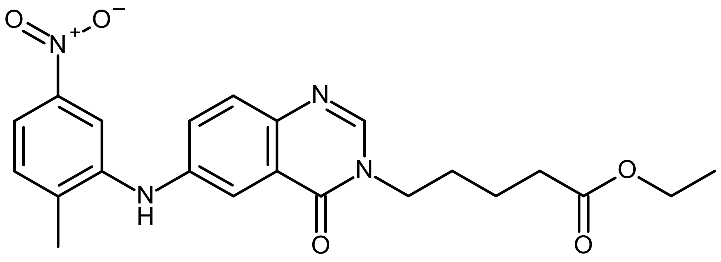

XantPhoS Pd G2 (1.006 g, 1.13 mmol) was added ethyl 5-(6-bromo-4-oxo-quinazolin-3-yl)pentanoate (5 g, 14.16 mmol), 2-methyl-5-nitroaniline (3.23 g, 21.23 mmol) and K2CO3 (4.89 g, 35.39 mmol) in 1,4-dioxane (100 mL) at 20 °C under nitrogen. The resulting mixture was stirred at 100 °C for 15 hours. The solids were removed by filtration and washed with THF. The solution was concentrated to afford crude product, which was purified by flash silica chromatography, elution gradient 0 to 3% MeOH in DCM. Pure fractions were evaporated to dryness to afford ethyl 5-[6-(2-methyl-5-nitro-anilino)-4-oxo-quinazolin-3-yl]pentanoate (5.40 g, 90 %) as a yellow solid. 1H NMR (300MHz, CDCl3) δ 1.28 (3H, t), 1.68-78 (2H, m), 1.82-1.92(2H, m), 2.36-2.41 (5H, m), 4.03 (2H, t), 4.11-4.18 (2H, m), 5.99 (1H, s), 7.36 (1H, d), 7.46-7.50 (1H, m), 7.69 (1H, d), 7.79-7.84 (1H, m), 7.98 (1H, s), 8.06 (1H, d); m/z (ES+), [M+H]+ = 425

Ethyl 5-[6-(5-amino-2-methyl-anilino)-4-oxo-quinazolin-3-yl]pentanoate

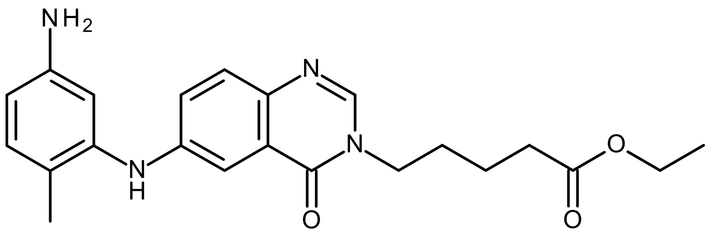

Ethyl 5-[6-(2-methyl-5-nitro-anilino)-4-oxo-quinazolin-3-yl]pentanoate (1 g, 2.36 mmol) and 10% Pd/C (0.125 g, 0.12 mmol) in MeOH (25 mL) were stirred under an atmosphere of hydrogen at 25 °C for 2 hours. The solids were removed by filtration and washed with MeOH. The solution was concentrated to afford ethyl 5-[6-(5-amino-2-methyl-anilino)-4-oxo-quinazolin-3-yl]pentanoate (0.850 g, 91 %) as a brown oil. 1H NMR (400MHz, CDCl3) δ 1.26 (3H, t), 1.70-1.76 (2H, m), 1.81-1.87 (2H, m), 2.15 (3H, s), 2.37 (2H, t), 4.00 (2H, t), 4.11-4.15 (2H, m), 5.69 (1H, s), 6.39-6.42 (1H, m), 6.66 (1H, d), 7.00 (1H, d), 7.34-7.36 (1H, m), 7.58 (1H, d), 7.64 (1H, d), 7.88 (1H, s); m/z (ES+), [M+H]+ = 395

Ethyl 5-[6-[5-[[3-(1-cyano-1-methyl-ethyl)benzoyl]amino]-2-methyl-anilino]-4-oxo-

quinazolin-3-yl]pentanoate

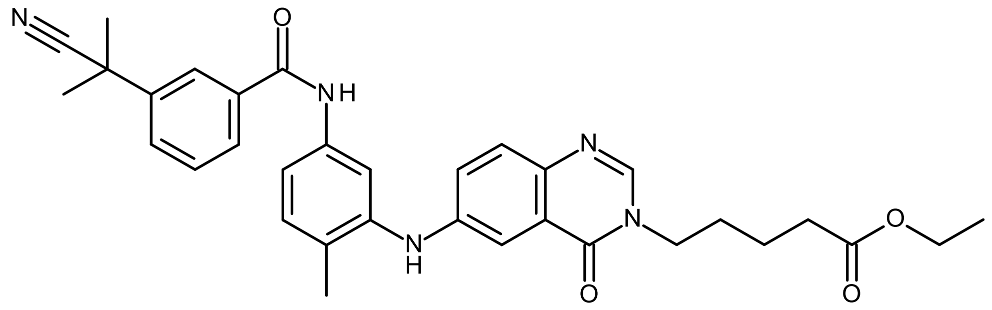

3-(1-cyano-1-methyl-ethyl)benzoic acid (0.489 g, 2.59 mmol) was added to ethyl 5-[6-(5-amino-2-methyl-anilino)-4-oxo-quinazolin-3-yl]pentanoate (0.85 g, 2.15 mmol), HATU (0.983 g, 2.59 mmol) and DIPEA (1.129 mL, 6.46 mmol) in DCM (15 mL) at 20°C. The resulting mixture was stirred at 25 °C for 5 hours. The reaction mixture was diluted with DCM (50 mL), and washed sequentially with saturated NaHCO3 (2x 25 mL), water (20 mL), and saturated brine. The organic layer was dried over Na2SO4, filtered and evaporated to afford crude ethyl 5-[6-[5-[[3-(1-cyano-1-methyl-ethyl)benzoyl]amino]-2-methyl-anilino]-4-oxo-quinazolin-3-yl]pentanoate (1.050 g, 86 %) as a brown oil which was used without further purification. m/z (ES+), [M+H]+ = 566

5-[6-[5-[[3-(1-Cyano-1-methyl-ethyl)benzoyl]amino]-2-methyl-anilino]-4-oxo-quinazolin-3-yl]pentanoic acid

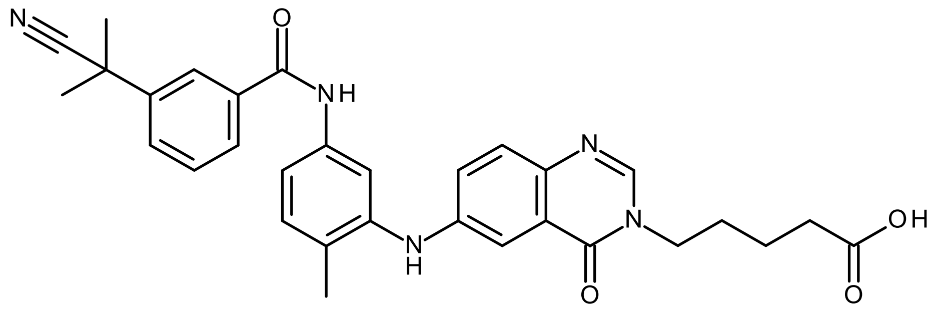

NaOH (9.28 mL, 18.56 mmol) was added to ethyl 5-[6-[5-[[3-(1-cyano-1-methyl-ethyl)benzoyl]amino]-2-methyl-anilino]-4-oxo-quinazolin-3-yl]pentanoate (1.05 g, 1.86 mmol) in THF (50 mL) at 20 °C . The resulting mixture was stirred at 25 °C for 15 hours. The aqueous layer was extracted with THF (3 x 100 mL). The organic layers were combined and washed with brine. The organic layer was dried over Na2SO4, filtered and evaporated to afford crude 5-[6-[5-[[3-(1-cyano-1-methyl-ethyl)benzoyl]amino]-2-methyl-anilino]-4-oxo-quinazolin-3-yl]pentanoic acid (0.750 g, 75 %) as a yellow solid. 1H NMR (400MHz, CDCl3) δ 1.46-1.57 (2H, m), 1.71-1.74 (5H, m), 2.19 (3H, s), 2.24-2.28 (2H, m), 3.98-4.06 (2H, m), 7.27 (1H, d), 7.40 (1H, d), 7.46-7.64 (5H, m), 7.73 (1H,

d), 7.79 (1H, d), 7.91-7.94 (2H, m), 8.02 (1H, s), 8.87 (1H, s), 10.28 (1H, s); m/z (ES+), [M+H]+ = 538

3-(1-Cyano-1-methyl-ethyl)-N-[4-methyl-3-[[4-oxo-3-[5-oxo-5-[[4-(1,2,4,5-tetrazin-3-yl)phenyl]methylamino]pentyl]quinazolin-6-yl]amino]phenyl]benzamide (AZ13-tet)

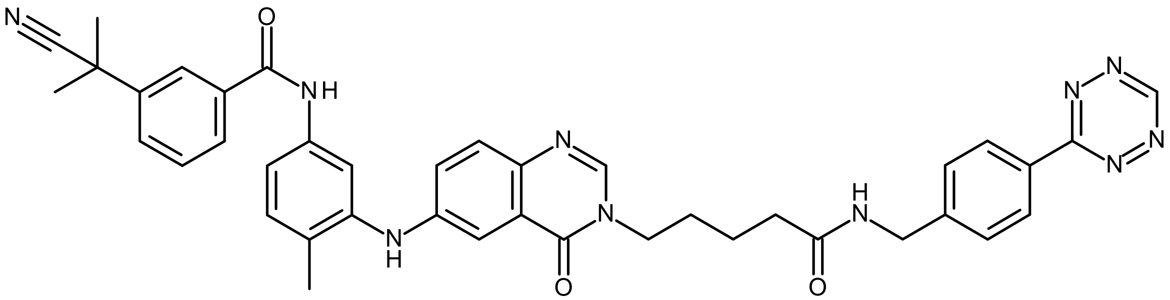

DIPEA (0.065 mL, 0.37 mmol) was added to 5-[6-[5-[[3-(1-cyano-1-methyl-ethyl)benzoyl]amino]-2-methyl-anilino]-4-oxo-quinazolin-3-yl]pentanoic acid (100mg, 0.19 mmol), [4-(1,2,4,5-tetrazin-3-yl)phenyl]methanamine hydrochloride (42.5 mg, 0.19 mmol) and HATU (106 mg, 0.28 mmol) in DCM (10 mL) at 20 °C . The resulting mixture was stirred at 25 °C for 15 hours. The reaction mixture was diluted with DCM (100 mL), and washed sequentially with 1M aqueous NaOH (50 mL), saturated brine (50 mL), and water (50 mL). The organic layer was dried over Na2SO4, filtered and evaporated to afford crude product. The crude product was purified by preparative HPLC. Column: XBridge Prep OBD C18 Column 30×150mm 5um;Mobile Phase A:Water(10 mmol/L NH4HCO3+0.1%NH3.H2O), Mobile Phase B: ACN; Flow rate: 60 mL/min; Gradient: 46% B to 51% B in 8 min; 254/220 nm; Rt: 7.48 min. Fractions containing the desired compound were evaporated to dryness to afford 3-(1-cyano-1-methyl-ethyl)-N-[4-methyl-3-[[4-oxo-3-[5-oxo-5-[[4-(1,2,4,5-tetrazin-3-yl)phenyl]methylamino]pentyl]quinazolin-6-yl]amino]phenyl]benzamide (AZ13-tet) (50.8 mg, 38.6 %) as a pink solid. 1H NMR (500 MHz, CDCl3) δ 1.67 – 1.73 (8H, m), 1.74 – 1.81 (2H, m), 2.21 (3H, s), 2.30 (2H, t, J = 7.1 Hz), 3.93 (2H, t, J = 7.0 Hz), 4.47 (2H, d, J = 5.9 Hz), 5.88 (1H, s), 6.56 (1H, t, J = 5.9 Hz), 7.16 (1H, d, J = 8.3 Hz), 7.28 – 7.35 (2H, m), 7.39 – 7.45 (3H, m), 7.50 (1H, d, J = 8.8 Hz), 7.57 – 7.65 (3H, m), 7.75 (1H, d, J = 7.9 Hz), 7.81 (1H, s), 7.96 (1H, s), 8.44 – 8.49 (3H, m), 10.14 (1H, s); 13C NMR (126 MHz, CDCl3) 17.61 (CH3), 22.75 (CH2), 28.90 (CH2), 29.09 (2x CH3), 35.65 (CH2), 37.26 (C), 43.28 (CH2), 46.16 (CH2), 110.43 (CH) , 112.77 (CH), 115.90 (CH), 123.08 (C), 124.31 (CH), 124.33 (CH), 126.27 (C), 126.52 (CH), 128.63 (2x CH), 128.67 (2x CH), 128.71 (C), 128.82 (C), 129.36 (CH), 130.65 (C), 131.62 (CH), 135.99 (C), 136.95 (C), 140.36 (C), 141.98 (C), 142.33 (C), 143.62 (CH), 143.84 (C), 144.22 (C), 157.84 (CH), 161.21 (C), 165.52 (C), 166.31 (C), 172.77 (C), 1x C not observed; m/z (ES+), [M+H]+ = 707; HRMS: Calculated for $C_{40}H_{39}N_{10}O_3$ 707.3205, found 707.3207, error 0.3 ppm
